# Recent Trends and Developments in Multifunctional Nanoparticles for Cancer Theranostics

**DOI:** 10.3390/molecules27248659

**Published:** 2022-12-07

**Authors:** Ali A. Rabaan, Rehab Bukhamsin, Hajir AlSaihati, Saleh A. Alshamrani, Jehad AlSihati, Hani M. Al-Afghani, Roua A. Alsubki, Abdulmonem A. Abuzaid, Saleh Al-Abdulhadi, Yahya Aldawood, Abdulmonem A. Alsaleh, Yousef N. Alhashem, Jenan A. Almatouq, Talha Bin Emran, Shamsah H. Al-Ahmed, Firzan Nainu, Ranjan K. Mohapatra

**Affiliations:** 1 Molecular Diagnostic Laboratory, Johns Hopkins Aramco Healthcare, Dhahran 31311, Saudi Arabia; 2 College of Medicine, Alfaisal University, Riyadh 11533, Saudi Arabia; 3 Department of Public Health and Nutrition, The University of Haripur, Haripur 22610, Pakistan; 4 Dammam Regional Laboratory and Blood Bank, Dammam 31411, Saudi Arabia; 5 Department of Clinical Laboratory Sciences, College of Applied Medical Sciences, University of Hafr Al Batin, Hafr Al Batin 39831, Saudi Arabia; 6 Department of Clinical Laboratory Sciences, College of Applied Medical Sciences, Najran University, Najran 61441, Saudi Arabia; 7 Internal Medicine Department, Gastroenterology Section, King Fahad Specialist Hospital, Dammam 31311, Saudi Arabia; 8 Laboratory Department, Security Forces Hospital, Makkah 24269, Saudi Arabia; 9 iGene Center for Research and Training, Jeddah 23484, Saudi Arabia; 10 Department of Clinical Laboratory Sciences, College of Applied Medical Sciences, King Saud University, Riyadh 11362, Saudi Arabia; 11 Medical Microbiology Department, Security Forces Hospital Programme, Dammam 32314, Saudi Arabia; 12 Department of Medical Laboratory Sciences, College of Applied Medical Sciences, Prince Sattam Bin Abdulaziz University, Riyadh 11942, Saudi Arabia; 13 Dr. Saleh Office for Medical Genetic and Genetic Counseling Services, The House of Expertise, Prince Sattam Bin Abdulaziz University, Dammam 32411, Saudi Arabia; 14 Department of Clinical Laboratory Sciences, Mohammed AlMana College of Health Sciences, Dammam 34222, Saudi Arabia; 15 Department of Pharmacy, BGC Trust University Bangladesh, Chittagong 4381, Bangladesh; 16 Department of Pharmacy, Faculty of Allied Health Sciences, Daffodil International University, Dhaka 1207, Bangladesh; 17 Specialty Paediatric Medicine, Qatif Central Hospital, Qatif 32654, Saudi Arabia; 18 Department of Pharmacy, Faculty of Pharmacy, Hasanuddin University, Makassar 90245, Indonesia; 19 Department of Chemistry, Government College of Engineering, Keonjhar 758002, India

**Keywords:** cancer therapy, nanotheranostics, plasmonic nanoparticles, drug delivery, nanomaterials

## Abstract

Conventional anticancer treatments, such as radiotherapy and chemotherapy, have significantly improved cancer therapy. Nevertheless, the existing traditional anticancer treatments have been reported to cause serious side effects and resistance to cancer and even to severely affect the quality of life of cancer survivors, which indicates the utmost urgency to develop effective and safe anticancer treatments. As the primary focus of cancer nanotheranostics, nanomaterials with unique surface chemistry and shape have been investigated for integrating cancer diagnostics with treatment techniques, including guiding a prompt diagnosis, precise imaging, treatment with an effective dose, and real-time supervision of therapeutic efficacy. Several theranostic nanosystems have been explored for cancer diagnosis and treatment in the past decade. However, metal-based nanotheranostics continue to be the most common types of nonentities. Consequently, the present review covers the physical characteristics of effective metallic, functionalized, and hybrid nanotheranostic systems. The scope of coverage also includes the clinical advantages and limitations of cancer nanotheranostics. In light of these viewpoints, future research directions exploring the robustness and clinical viability of cancer nanotheranostics through various strategies to enhance the biocompatibility of theranostic nanoparticles are summarised.

## 1. Introduction

Due to limitations in the early identification and diagnosis of solid internal tumours, effective cancer therapy has been limited for many years. Standard chemotherapy for cancer also kills normal bystander cells and has a detrimental effect on the illness outcome. In the past several decades, numerous strategies have been developed for the targeted delivery of medications to cancer cells, including liposomes, nano-delivery systems, and antibody-conjugated drug delivery systems. The success of these strategies varies, depending on the type of cancer being treated. However, the early identification or diagnosis of cancer, followed by targeted medication administration, is still the most sought-after method for the effective treatment of cancer.

Recently, considerable attention has been paid to advancing innovative diagnostic techniques for more efficient and successful cancer treatment. Previously, recent advances in imaging techniques have led to nanoparticle-based cancer detection, which has changed cancer diagnosis. In the past, nanostructure formulations have been approved and widely used to supplement conventional chemotherapy in cancer patients. However, recent advances in employing these formulations for significant therapeutic and diagnostic purposes (theranostics) have considerably enhanced the treatment of cancer patients [1]. Theranostic NPs are nanoscale diagnostic and therapeutic systems that are biocompatible, biodegradable, and multifunctional. Numerous diseases, including cancer, diabetes, and infectious diseases, have been managed on an intuitive level with the use of these technologies [2]. The ideal characteristics, namely, (i) biocompatibility, (ii) targeted accumulation in tissues of interest, (iii), the unravelling of morphological and biochemical scenarios under disease conditions, (iv) targeted drug delivery, and (v) the ability to be metabolised into non-toxic by-products, should be demonstrated with NP-based theranostics. Additionally, selective tumour targeting can be achieved by conjugating NPs with ligands specific to oncogenic (overexpressed in cancer) receptors such as folate and integrin. Other receptors include Prostate Specific Membrane Antigen (PSMA) and Urokinase Plasminogen Activator Receptor (UPAR). Moreover, the NPs can be conjugated with siRNAs against these receptors and a fluorescent dye to report their specific binding [3].

### 1.1. Working Principle of Cancer Nanotheranostics

Nanotechnology has gained attention in the therapeutic and diagnostic domains, as it works on targeting a specific site. In therapeutics, drug molecules, including small drugs, peptides, and nucleic acids, either encapsulate or bind with the nanomaterials. This forms a nano-sized therapeutic entity that targets the cancerous cells without attaching to the healthy cells. After targeting the cancerous cell, the therapeutic entities from the nanoparticle are released to the site and perform the therapeutic action. Similarly, in diagnostics, nanoparticles are designed to identify tumour cells. Here, differently shaped nanoparticles are formed, which include nanotubes and nanoshells. The antibody is attached to a nanoshell, recognises the tumour cell and gives the signal. The antibody indirectly binds to the nanoparticle, first linking to polyethylene glycol (PEG) and then to the nanoparticle.

#### 1.1.1. Metallic Nanoparticles for Cancer Theranostics

Metallic nanoparticles have unique physicochemical properties and are the ideal materials for the therapeutic targeting of diseases such as cancer. They can be synthesised using several methods, including wet chemical synthesis, pyrolysis, hydrothermal process, precipitation, co-precipitation, sol–gel procedure, microemulsion, sonolysis, and reduction.

The physical properties, such as fluorescence, luminescence, and surface plasmon resonance, along with the chemical properties, including the augmentation of enzymatic activity, are preferred when designing targeted therapeutics. Additionally, these nanoparticles exhibit a high ratio of surface area to volume, enhancing their likelihood as suitable candidates to be coated with various drugs and small molecules. The surface functionalization of these nanoparticles makes them preferred for targeted cancer therapeutics, leading to reduced adverse effects. Furthermore, metal nanoparticles have been implicated in cancer imaging and diagnosis [4,5,6]. For instance, metallic nanoparticles with paramagnetic/superparamagnetic/ferromagnetic features can be used for imaging cancer tissue. The photoluminescent ability of these NPs can create reactive nitrogen and reactive oxygen species that could be responsible for killing cancerous cells [7,8].

**Figure 1:**
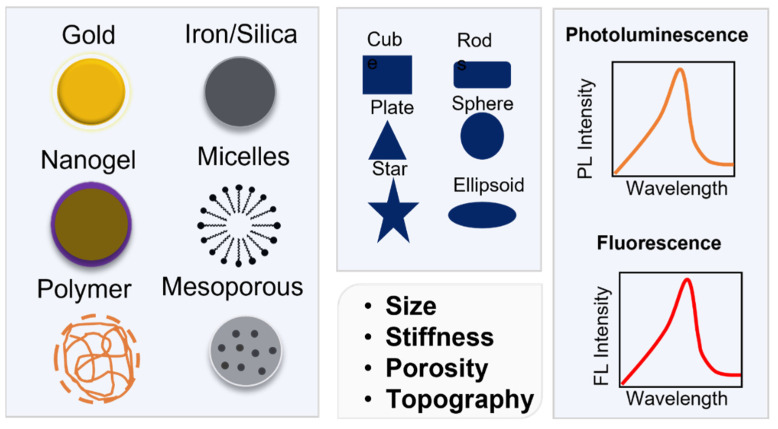
Physicochemical characteristics of nanoparticles.

Furthermore, the nanoparticle-induced destruction of cancer cells can be attributed to other phenomena, including hyperthermia and the photothermal effect. The ability of NPs to induce these processes depends on the topographical features, form, and morphology of the nanoparticles. Metal NPs are biocompatible, have inherent anticancer potential, do not accumulate in the body, and can be modified to be encapsulated in conjugates with other NPs. For imaging purposes, they can also be various fluorescent dyes and radioisotopes [9]. Figure 1 summarizes different types of metallic nanoparticles used for cancer theranostic.

#### 1.1.2. Types of Metallic Nanoparticles in Cancer Nanotheranostics

Metallic nanoparticles are directly hazardous to live cells, but encapsulating these NPs into host biofilms makes them useful for cancer therapy and diagnostics. Gold (Au) is widely used in cellular imaging for diagnostic purposes. Gold is also a plasmonic NP, and it is used in photothermal therapy to destroy brain tumour cells. Silica (Si) is used as a drug carrier and for gene delivery. Iron (Fe) has magnetic properties; thus, it is used in magnetic imaging for cancer diagnostics. Silver (Ag) NPs are used in radiation therapy for cancer, either independently or in combination with Fe_3_O_4_. The various methods for synthesising metallic or metalloid NPs are summarised in Table 1.

### 1.2. Application of Metallic Nanoparticles in Cancer Theranostics

Metal nanoparticles are used for numerous biomedical applications, including anticancer applications, radiotherapy enhancement, drug delivery, thermal ablation, antibacterial applications, diagnostic assays, antifungal applications, and gene delivery. They are functionalized with various functional groups, including peptides, antibodies, RNA, DNA, and potentially biocompatible polymers, to target distinct cell types. For instance, a nanostructure composed of branching gold shells was employed to treat breast cancer. In addition, magnetic nanoparticles were also used to treat cancer cells. In cancer theranostics, metal nanoparticles are the most widely employed agents. They have several benefits over traditional cancer treatment, including fewer side effects and a decreased incidence of drug resistance. Recent modifications of these NPs with aptamers, silica, DNA, photosensitizers, photoluminescence, and fluorescent molecules have made them more suitable for imaging, diagnostics, and therapy [13,14].

**Figure 2:**
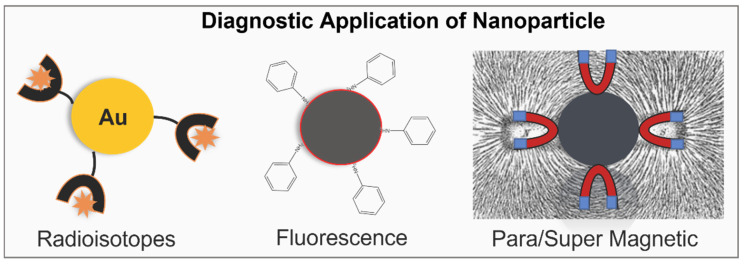
Surface modifications of metallic/metalloids NPs for cancer imaging (diagnostics).

**Figure 3:**
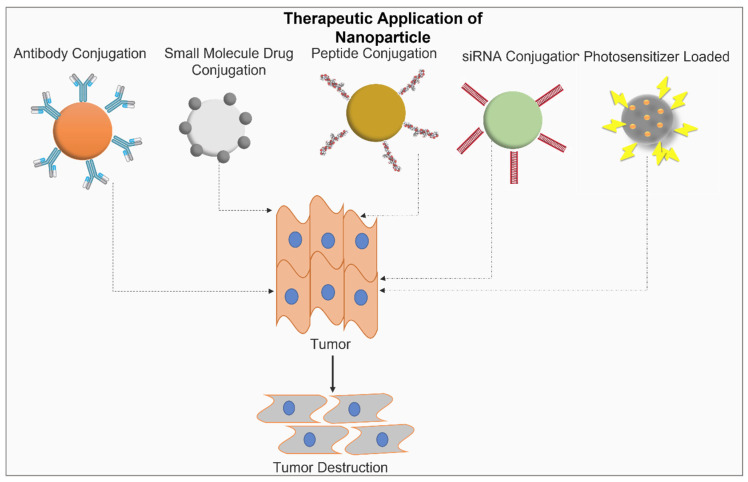
Surface modification of metallic/metalloid NPs for cancer therapeutics.

These modifications are summarised in Figure 2 and Figure 3. Figure 2 mentions the surface modifications of NPs for imaging (diagnostic) purposes. Radioisotopes are bound to the Au-NPs that are used to label the targeted site. Similarly, fluorescence chemical groups are bound with nanoparticles so that the nanoparticle can be easily identified when it binds with the target cell. As shown in Figure 2, superparamagnetic iron nanoparticles are the type of magnetic nanoparticle that offers magnetic properties in the presence of an external magnetic field and is used for imaging. Figure 3 shows the surface modifications of NPs for therapeutic purposes. Therapeutic antibodies, including small molecules, peptides, antibodies, siRNA, and photosensitizers, are attached to the nanoparticles and targeted to the cancerous cell. They target and destroy tumour cells. Several such preparations of modified metal NPs are currently in clinical trials and not yet approved. Through extensive laboratory studies, these modified metal NPs need to be pre-optimized in terms of stability, dosage, preparation method, and side effects. Once these conditions are satisfied, they can be used as a vital cancer-fighting medicinal tool. Moreover, the study conducted by Li et al. (2021) constructed peptide-conjugated metal clusters as catalytic antibodies, which work as biomarkers for specific diagnosis and treatment [15].

## 2. Various Plasmonic Nanoparticles and Their Application in Cancer Theranostic

### 2.1. Gold Nanoparticles

Gold nanoparticles (AuNPs) can absorb light and transform it into heat using a non-radioactive process [16,17,18,19,20]. This generated heat can then be transferred into the surrounding environment. For this photothermal therapy (PTT), a continuous-wave laser is used, having an absorption spectrum overlapping with that of AuNPs [21,22]. However, this treatment is generally suitable for superficial tumours, such as skin tumours [21,23]. In PTT, nanoparticles (the photothermal conversion agent) are accountable for the transformation of light into heat. Thus, they operate as nanosources of heat to raise the local temperature. In addition, PTT is used in the synthesis of the optical characteristics of several nanoparticle types, including gold nanoparticles [24]. In a recent study, antibodies against the EGFR receptors, conjugated onto AuNPs, induced PTT in carcinoma cells, leading to the inhibition of the tumour [25]. In another study, the coating of HA, a ligand for CD44, onto the surface of Au nanocages resulted in the specific recognition and targeting of cancer cells with overexpression of CD44 [26]. These HA-conjugated Au nanocages can be preloaded with doxycycline. They are taken up in the cells via the process of receptor-mediated endocytosis and are degraded inside the lysosomes, leading to the release of doxycycline. DOX-loaded, HA-conjugated Au NPs inhibited tumour growth, and when combined with PTT, they resulted to be complete tumour inhibitors. Moreover, ROS-mediated gold nanocages (AuNCs) with PEG initiated tumour cell apoptosis [26,27]. In another study, the lipid HB-AuNC combination was developed for in vitro two-photon photothermal cancer treatment. The combination of photosensitizers and photothermal transducers as well as the use of the two-photon methods resulted in one-time administration and irradiation for antitumor therapy [28]. In addition, the study conducted by Gao et al. (2015) constructed a peptide AuNP nanoprobe to quantitatively determine the GPIIb/IIIa on the cell membrane, which works as a biomarker to identify the relevant diseases [29]. Table 2 shows the types and applications of Au NPs.

### 2.2. Zinc Nanoparticles

Zinc oxide nanoparticles (ZnO NPs) are semiconductors in nature with an intrinsic photoluminescent nature. The former property can be employed to generate reactive oxygen species (ROS). The latter is more applicable to biosensors [8,30]. ZnO NPs are generally biocompatible, making them the preferred choice for drug delivery. They have the unique characteristic of being inherently cytotoxic against cancer cells. This effect is because of their inherent semiconductor property, which leads to the generation of ROS and thus the killing of cancerous cells [30].

In the case of semiconductors, electrons in certain bands possess energy, leaving void bands (band gaps) in between. On the other hand, metals have continuous electronic states with no void gaps, as in the case of ZnO. This void gap is approximately 3.3 eV. The valence band of crystalline ZnO is left with vacant electron locations when UV light is incident upon it because the electrons are promoted to the conduction band when the light strikes it. These promoted electrons and empty electron positions, or holes, then travel up to the NP surface and react with OH- ions and oxygen, respectively [8]. In the context of ZnO NPs, numerous electron holes are present without UV stimulation, making them less conductive. Thus, in ZnO NPs, the size is inversely proportional to the quality of the NPs. Defects in the nanocrystals create more electron holes, which leads to ROS production [31]. ROS, in turn, trigger a signalling cascade, causing irreversible cellular damage due to oxidative stress and eventually leading to cell death. Table 3 shows the applications and types of ZnO NP in cancer treatment.

### 2.3. Silver Nanoparticles

Silver nanoparticles (AgNPs) can scatter and absorb some of the light incident on them. This light may then be used for the targeted destruction of cancer cells once it has been absorbed. The selective penetration of AgNPs can be achieved by coating the surfaces with tumour-specific ligands. On the other hand, scattered light can be employed for cancer imaging among the metals that exhibit the property of plasmon resonance, namely, Cu, Ag, and Au. Here, Ag NPs show the maximum efficiency due to the equal number of positive and negative ions. These plasmons combine with visible light to display SPR [32]. Ag NPs, upon selective entry into cancerous cells, impair the activity of the proteins required to neutralise ROS, such as thioredoxin and glutathione. This leads to an accumulation of ROS that initiates an inflammatory response, leading to mitochondrial damage. Once the mitochondria are damaged, apoptosis-inducing factors are released, leading to programmed cell death [33]. Table 4 shows the applications and types of AgNPs in cancer treatment.

### 2.4. Iron Nanoparticles

Iron is the most widely implicated metal in cancer therapeutics. Iron oxide nanoparticles (FexOn NPs, where x = 1, 2, and 3, and n = 1, 3, and 4), defined as FeNPs, have been used for cancer diagnosis, imaging, and therapeutics. They are widely used for liver imaging (MRI) to enhance contrast. These NPs can be implicated as drug carriers through drug conjugation at the NP surface, i.e., covalent conjugation, and via entrapment in the polymeric matrix. Many studies have shown the conjugation of cancer drugs on these NPs, such as doxorubicin, methotrexate, and paclitaxel [39,40,41].

Recently, iron oxide NPs conjugated to methotrexate and chlorotoxin were used to target cancer therapeutics. In this complex, chlorotoxin acted as a targeting ligand, while methotrexate worked as a therapeutic agent. The complex showed better cytotoxic effects towards tumour cells and exhibited theranostic applications [42]. In other studies, FeNPs coated with monoclonal antibodies were used to detect ovarian cancers overexpressing mucin-1 (MUC-1). These NPs showed better accumulation inside the tumour and exhibited faster tumour detection without any toxic effects [43]. Folic acid was shown to be conjugated in another investigation, and FeNPs showed specificity for detecting breast cancer cells [44]. Table 5 shows the applications and types of Fe-NPs.

**Figure 4:**
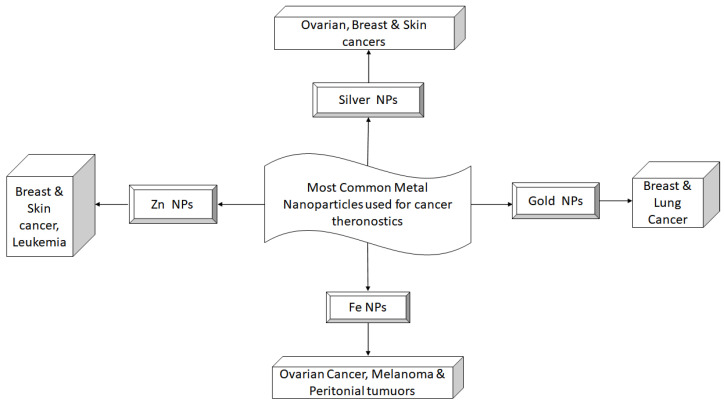
Summary of metallic nanoparticles used in various cancer types.

Additionally, aptamers can be coupled with FeNPs to target hepatocellular cancer in a particular manner for imaging and medicine administration purposes. The aptamer, in this case, is specific to the DNA of the molecule for the adherence of epithelial cells [45]. These NPs have also been implicated in the delivery of macromolecules such as DNA, proteins, and peptides. For the delivery of these macromolecules, NPs are coated with positively charged polymers, such as dextran, chitosan, and polyethyleneimine. Recently, nanocomposites of polycation and iron oxide have been used as siRNA carriers that are visible through MRI and can combat the multidrug resistance phenotype [46]. It has been shown that the generation of telomerase in hepatocellular carcinoma cells may be suppressed by the conditional release of siRNA in response to deteriorating conditions inside the cells. This finally leads to the death of the cell [47]. The various types of metallic nanoparticles and their theranostic applications against different cancer types are summarized in Figure 4. Apart from the traditional and most widely used metal NPs, several recent advancements have led to the synthesis of NPs that are better suited for cancer theranostics. These are discussed in the following sections.

**Table 5:** Types of FeNPs and their applications in cancer therapy.

Therapeutic Entity	Type of Fe NP	Application	References
Magnetic hyperthermia (MHT)	Superparamagnetic iron oxide nanoparticles	Normal cell restoration after cancer cell destruction	[48]
OVA	Fe_3_O_4_-OVA	Tumour inhibition	[49]
poly(lactic-co-glycolic acid) (PLGA) and chlorin E6 (Ce_6_)	Fe_3_O_4_-PLGA-Ce_6_	Tumour cell ferroptosis	[50]
DOX	DOX-Fe_3_O_4_	Tumour lymph node detection and therapy	[51]
Doxorubicin–Gelatin/Fe_3_O_4_–Alginate	DG/FA NPs	Targeted drug delivery and cancer therapy	[52]

### 2.5. Chalcogenide Nanoparticles

#### 2.5.1. Chalcogens

Elements such as selenium, tellurium, sulphur, polonium, oxygen, and livermorium have usually been linked with chalcogens [53]. Sulphur, selenium, and tellurium are the metalloids that have been explored the most as nanocomposites with cancer treatment potential. Common applications of sulphur and compounds containing sulphur are fertilisers, antimicrobials, and antifungal compounds. On the other hand, sulphur has fascinating nanoscale characteristics, including biodegradability, safety, and biocompatibility, which play vital roles in catalytic bioremediation, as well as in antibacterial and anticancer compounds [53,54,55,56,57]. Selenium (metalloid), with chemopreventive characteristics, also serves as a regulating element in the body. Chalcogens such as sulphur, tellurium, and selenium react chemically with other metals to generate chalcogenide NPs at the nanoscale. According to how many distinct chalcogens they contain, they are classified as mono-, di-, or polychalcogenides. Binary and ternary nanocrystals were also identified in chalcogenides. Nanoclusters, NPs, and quantum dots are all structural forms of chalcogenide nanoassemblies [58,59]. The chemical synthesis of NP involves the use of various processes, including the polymerization of monomers, the dispersal of prepared polymers, and ionic gelation. Various chalcogens and chalcogenides have demonstrated exceptional physicochemical and pharmacological qualities that are important in cancer prevention and treatment [60,61]. Moreover, Table 6 shows the chalcogenide nanoparticles used in cancer treatment.

#### 2.5.2. Selenium

Antimicrobial, photocatalytic, antioxidant, and anticancer properties have been reported for green selenium nanoparticles (SeNPs). Their antioxidant properties are connected to their ability to sequester Se at the release site of reactive oxygen species (ROS), thus inhibiting the generation of free radicals that cause DNA oxidative stress [68]. SeNPs with smaller sizes have superior scavenging action than more significant NPs, owing to the central significance of NP size in free radical scavenging [69]. The SeNP capacity to form bonds with metal ions and proteins found inside the cell has been related to their anticancer action. SeNPs, for example, interact and bind with Cu^2+^ and DNA to create a ternary complex, reducing Cu^2+^ to Cu^+^, which is then re-oxidized to produce reactive oxygen species that cause cell apoptosis. A cancer-specific apoptosis mechanism has been reported, because copper (Cu) ions (found to be present in plenty of cancer cells) are required for the generation of free radicals that cause oxidative damage [70]. After being exposed to SeNPs, human melanoma (A375) cells exhibited cellular oxidative stress and mitochondrial malfunction. This finding demonstrates that SeNPs react with intracellular proteins participating in mitochondrial and glycolytic activities. This was the case before induced apoptosis occurred [71].

#### 2.5.3. Tellurium

Tellurium nanoparticles (TeNPs) (synthesised as nanodots, nanorods, nanowires, and nanocubes) exhibit antibacterial, antioxidant, and cancer-fighting properties [72,73,74,75]. Compared with their chemically derived counterparts, biogenic NPs have the added benefit of selective toxicity. The cytotoxicity of TeNPs may be linked to their potential to bind DNA and cellular proteins, which results in oxidative damage and DNA degradation and ultimately leads to cell apoptosis via mitochondrial pathways. Furthermore, the biocompatibility of biogenic TeNPs may be influenced by the biomolecule capping type [74,76]. TeNPs can be produced using citrus fruit extracts; orange extract (OR-TeNPs) and lemon extract (LEM-TeNPs) were compared in a comparative study [62]. Research on biogenic TeNPs’ anticancer properties is limited, but it is intriguing and worth investigating.

#### 2.5.4. Sulphur

By controlling redox imbalances, sulphur has been demonstrated to have an oncoprotective effect on several bioactive chemicals in plants [77,78]. Its cytoprotective antioxidant capacity has also been shown in mouse models. Its existence in amino acids such as cysteine and methionine, as well as the production of disulphide connections in tertiary protein structures, might explain this [79]. These amino acids have also been demonstrated to perform free radical scavenging [80]. Methionine has been identified as an antioxidative barrier in several proteins, where it is easily oxidised and crucial to the oxidative stress repair pathway. As a result, they function as endogenous antioxidants within cells [81]. Sulphur nanoparticles (SNPs) were the first to show an anticancer effect on oral cancer cells by inducing apoptosis. Even though the cause of SNP cancer cell cytotoxicity is still unknown, three cytotoxic processes have been linked to SNP anticancer properties: ion-dependent oxidative damage, membrane permeation, and cell cycle arrest induction of apoptosis [82].

#### 2.5.5. Cadmium

**Figure 5:**
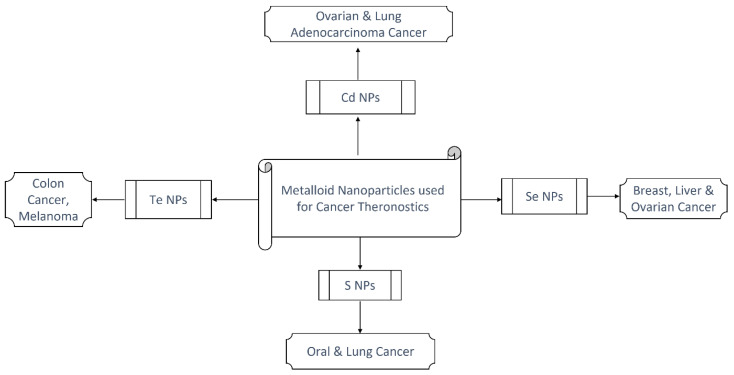
Summary of chalcogen nanoparticles with their therapeutic usage.

Cadmium sulphide quantum dots (CdS-QDs) have been used to induce cellular oxidative stress in photodynamic treatment for treating cancer cells. Cd-based chalcogenides, such as CdSe and CdTe, have shown anticancer properties through processes comparable to CdS [83,84,85]. CdTe QDs link to serum proteins and pass through the cellular membrane through clathrin-mediated endocytosis. They are destroyed in the lysosomes after being incorporated into the cells. Cd^2+^ is discharged, causing mitochondrial augmentation and also causing cancer cells to die by inducing internal and extrinsic apoptosis [86]. The lethal action of CdSe QD in A549 cells has also been shown to be mediated by ROS-induced DNA damage, which results in the induction of apoptosis [87]. Photodynamic therapy (PDT) for treating cancers has also been used with QDs. QDs absorb photons of a specified wavelength and form excitons (e- holes). Their energy is subsequently transmitted to nearby species or molecular oxygen, resulting in singlet (no net magnetic momentum) oxygen radicals that cause cell damage [88]. The various types of metalloid/chalcogenide nanoparticles and their implications across multiple cancer types are summarized in Figure 5.

## 3. Silica Nanoparticles

Among the different types of functional NPs, silica (SiO_2_) NPs possess distinctive structural and functional characteristics. In the context of light-based nanomedicines for the imaging and therapeutics of cancer and other diseases, SiO_2_ NPs have shown significant potential. SiO_2_ NPs that are mesoporous and non-porous have excellent light-absorbing abilities in the visible and near-infrared regions. These optical properties make them suitable for in vivo imaging even at the nanoscale. Additionally, SiO_2_ NPs have shown tremendous potential in combining light-based diagnosis with therapeutics, such as photo-theranostics.

SiO_2_ NPs can be classified into inorganic and organic types based on their precursors’ synthesis methods. Inorganic NPs do not contain any carbon molecules, although they may have been synthesised from carbon-containing precursors such as alkoxysilanes. In contrast, organic silica NPs are further classified into “organically modified silane NPs”, or ORMOSIL, and functional organosilica NPs. While ORMOSIL NPs do not contain any functional groups, functional organosilica NPs do contain functional groups, i.e., epoxy and thiol groups. These functional organosilica NPs can be synthesised using organosilane reagents with functional groups [89,90,91,92]. Inorganic silica NPs conjugated with aptamers have been used for various applications, such as extraction and fluorescent labelling in acute lymphoblastic leukaemia cell lines, breast carcinoma cell lines, and Burkitt’s lymphoma cell lines [93,94,95].

On the other hand, ORMOSIL NPs coated with Rhodamine B and conjugated to bioactive molecules such as monoclonal antibodies are used to target drug delivery specifically to pancreatic cancer cell lines. The conjugation of NPs with bioactive molecules enhances the uptake efficiency of these NPs [96]. In addition, SiO_2_ NPs containing photosensitizers such as protoporphyrin IX and hypocrellin B are excellent tools for photodynamic therapy, or PDT [97,98,99], in tumour tissues. Table 6 shows the chalcogenide nanoparticles used in cancer treatment.

**Figure 6:**
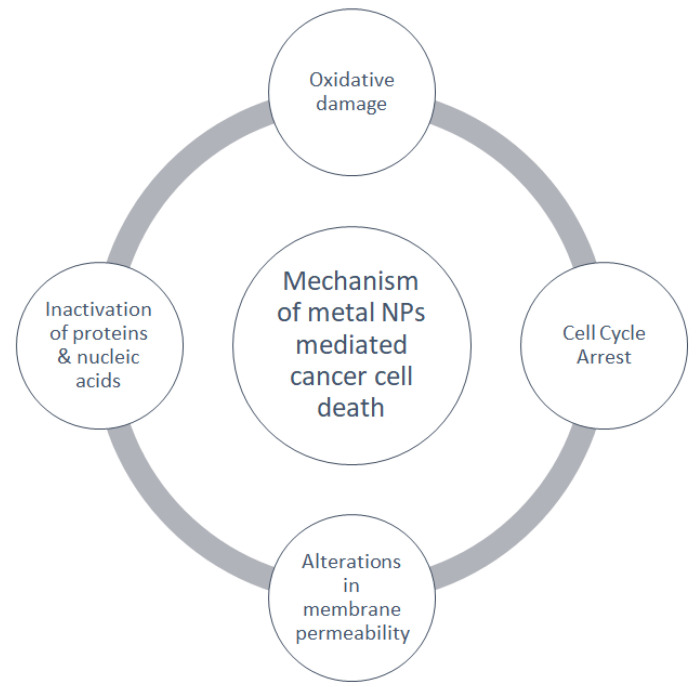
Mechanism of metal nanoparticle-mediated cell death.

Overall, the mechanical actions by which metallic or metalloid NPs induce cancer cell death are summarized in Figure 6.

## 4. Hybrid Nanoparticles

### 4.1. Magnetic NPs

Magnetic nanoparticles (MNPs) have various uses in biomedicine, including medication administration, auxiliary evaluation and assessment, and therapy [100]. In a high-frequency magnetic field, MNPs have a magnetocaloric action, which can eradicate tumour cells indirectly [101]. They are made of nickel, iron, cobalt, and some other metals, as well as their oxides. The diameter of superparamagnetic MNPs is predominantly that of superparamagnetic iron oxide nanoparticles (SPIONs), <50 nm [102]. MNPs are magnetically non-permanent. They show magnetism in an externally applied magnetic field and are primarily employed to research the role of MNPs in vivo. MNPs have highly definite surface areas and can contain a range of tiny proteins, molecules, RNA, and other compounds [103,104]. These characteristics make it simpler to enrich and sort them and to move and detect them in different directions. The following sections discuss various modifications of the basic magnetic NPs for specific applications.

### 4.2. Silica-Coated Magnetic Nanoparticles

Silica can be coated onto the surface of magnetic NPs (MNPs) [105,106,107,108]. Moreover, SiO_2_-coated MNPs can also be doped with Mn^2+^ to be cast off as a contrast agent for magnetic resonance imaging (MRI) [109,110]. Similarly, NPs of other metals can also be coated with silica to develop multifunctional NPs. The hydrophobic core of IONPs can be coated with silica using a micro-emulsion method, leading to the generation of silica NPs that can be detected using MRI [111]. Inhalation, topical skin penetration, and injection are all options for introducing nSiO_2_ microspheres into organisms. When inhaled, the nSiO_2_ microsphere drug-carrying mechanism crosses the barrier between lungs and blood to enter the bloodstream directly, allowing systemic delivery to be performed [112]. Iron oxide NPs encapsulated in nSiO_2_ microspheres may have their surfaces changed with -OH, -COOH, or -NH_2_ to make them active and persistent to react, resulting in a new type of silica nanoparticle that can chemically connect with proteins and increase its application range [113]. Simultaneously, because of the magnetic silica nanoparticle small particle size, large definite surface area, and potent magnetic and adsorption reactions, if active groups on the surface can reattach functional polymers or small molecules, they can turn out to be a versatile drug carrier substance with high performance.

### 4.3. Vesicle-Type Magnetic Nanoparticles

The framework of vesicle-type magnetic nanoparticles is a phospholipid bilayer with MNPs scattered inside. MNPs in liposomes range in dimension from 1 nm to 10 nm. They do not clog and discharge smoothly from the body due to their tiny size. They are made by encapsulating superparamagnetic magnetic nanoparticles in lipid unilamellar vesicles using size exclusion chromatography [114]. MNPs can be distributed into hydrophobic nanomagnetic liposomes (MNPs enclosed in a phospholipid bilayer), hydrophilic MNPs (liposomes with hydrophilic magnetic nanoparticles in the inner water core), or magnetic nanoparticles implanted on the phospholipid membrane surface [115]. Due to their biocompatibility and low toxicity, they are also used as biomedical magnetic resonance contrast agents, drug carriers, and hyperthermia intermediaries.

### 4.4. Polymer-Coated Magnetic Nanoparticles

Self-assembly and chemical covalent bond modification are two approaches for making magnetic polymer drug carriers [116]. The most significant research and application barrier is that magnetic nanoparticles are vulnerable to accumulation due to their high surface energy and distinct surface area, which makes uniform dispersion in polymers challenging. Several studies have changed the active groups (sulfo and amino groups) present on the membrane of magnetic nanoparticle carriers and packed biomolecules, along with stimulus-responsive and functional capabilities on the carrier, which depend on the MNPs and modify the polymer. On this basis, a responsive (quick to respond) magnetic polymer drug-carrying system was built using a set of intelligent nano-drug-controlled release mechanisms [116,117,118].

### 4.5. Super-Magnetic Iron Oxide Nanoparticles

Iron oxide, namely, Fe_3_O_4_ and Fe_2_O_3_, is a significant component of magnetic nanoparticles. Super-paramagnetism and coercive force are seen under a condition where the magnitude of iron oxide nanoparticles is less than a particular threshold point according to the limit value and saturation magnetization is lowered [119]. Thermal decomposition and co-precipitation, laser pyrolysis, microemulsions, and sol–gel are some processes that are used to make nanoscale metal cores [120]. Clinical trials have employed SPION formulations because they are nontoxic and biocompatible and also have good paramagnetic characteristics [121]. They can also bind to haemoglobin via regular physiological and metabolic routes, preventing build-ups inside the body. It can help people with weak renal and liver functions. SPIONs may also increase or decrease the intensity of T2WI signals [122]. They have significant utility in tumour detection, therapy, and disease monitoring. Moreover, various hybrid nanoparticles and their application in cancer therapy defined in Table 7.

## 5. Applications of Magnetic Nanoparticles in Theranostics

### 5.1. Drug and siRNA Delivery

MNPs with magnetizable implants or external magnetic fields can transport and attach elements at local locations in the magnetic drug targeting (MDT) technique, allowing the drug to be delivered remotely. Due to their tiny diameter, Fe_3_O_4_ MNPs have low toxicity, steady performance, high sensitivity, and simple access to raw components [131,132]. The quantity of iron in carriers is less than the total iron in anaemic patients’ supplements, which is harmless, and any excess iron in the body may be eliminated through the skin, bile, kidneys, and other organs. By slowing the drug loss and half-life during drug administration, the practicality of utilising iron oxide magnetic nanoparticles for targeted drug delivery is increased, and the medication time and efficiency are enhanced [126,131,133]. According to a study, doxorubicin (DOX) packing and folic acid transformation on SPIONs significantly improved DOX@FASPIONs in MCF-7 cells in vitro, and the mouse xenotransplantated MCF-7 breast tumour development inhibition effectiveness of DOX@FASPIONs showed high r2 relaxation (81.77 mM-1S-1) and no toxic effects on mouse organs after 35 days of treatment [134]. In a different study, DOX-containing heparin superparamagnetic iron oxide (DH-SPIO) nanoparticles were observed to be more effective than DOX in preventing tumour development and extending the rate of survival of tumour-bearing mice in vivo. The degree of pathological injury to cardiac tissue in the mice given DH-SPIO nanoparticles remained much smaller than that in animals given a similar dose of free DOX, suggesting that DH-SPIO nanoparticles have the potential to be used in drug combination treatment and clinical imaging [135]. Morever, in this section it was attempted to define numerous types of magnetic nanoparticles their drug delivery systems, and their use for the treatment of various forms of cancer as shown in Table 8.

### 5.2. Magnetic Hyperthermia

Magnetic hyperthermia induced by an external magnetic field has been shown to reduce cancer cells and boost the efficacy of other therapies. Because of its infinite tissue penetration capabilities and low risk of skin infection, magnetic hyperthermia is far more capable of translation than laser photothermal treatment. In a patient with cholangiocarcinoma excision, chemotherapy (gemcitabine/cisplatin) coupled with magnetic hyperthermia was effective. The patient demonstrated no advancement of cholangiocarcinoma on computerised tomography (CT) after 32 cycles of combination therapy, with no significant consequences within 4 months. Magnetic hyperthermia induced by ion beams can elevate the tumour’s core temperature to 40 °C, limiting tumour development [105,106,111,141,142,143,144]. Table 9 shows the applications and types of magnetic hyperthermia nanoparticles.

### 5.3. Magnetic Nanorobots

Nanobots can aid cancer therapy by selectively delivering therapeutic drugs to tumour blood vessels, performing circulatory diagnostics, advanced surgery, and tissue regeneration while lowering operation and rehabilitation time [151]. Magnetic nanorobots can also be used to remotely manage magnetic nanobots in the body for medicine administration or improved resonance imaging. When they enter the bloodstream, they can target particular malignant tumour cells and treat them using the in-built computational resources. This can reduce radiation and chemotherapy adverse effects while allowing more accurate medication administration and therapy to be performed [152]. Magnetic robots work against cancer using nanotheranostic technology, as described in Table 10.

### 5.4. DNA-Functionalized NPs

In opposition to free DNA molecules, DNA-NPs are created by grafting DNA molecules with a thiol end on the AuNP surface, resulting in distinct features, such as subsequent abrupt melting transitions and cooperative binding, as well as resistance to nuclease destruction [155,156,157]. The central core can be then substituted with numerous polymeric (Pd, Ag, Fe_3_O_4_, nanoshells, quantum dots, polymers, and proteins) and inorganic materials with different optical, catalytic, and physicochemical capabilities [158]. The core of DNA-NPs is emptied and coated with a single-stranded DNA shell to increase biocompatibility [159,160]. A technique for grafting DNA onto lanthanide-doped up-conversion nanoparticles was demonstrated in a study [161]. Direct DNA-metal ion coordination produced a sphere-shaped metal-DNA nanostructure for targeted drug therapy [162]. Similarly, DNA-lipid/polymer ampholytes have been studied for their ability to self-assemble nanostructures with protruding DNA molecules [163,164]. Furthermore, DNA hybridization-based techniques for integrating DNA nanostructures (DNA-NSs) and DNA-functionalized nanoparticles (DNA-NPs) into a unified nanoscale structure with enhanced optical characteristics and unique capabilities have been developed [165].

The negatively charged DNA layer has improved nucleic acid stability and a distinct structure that is thought to prevent enzymatic nucleic acid breakdown by endogenous nucleases and the innate immune response [155,166]. Patel et al. found that serum nucleases and dicers had a lower preference for moderate duplexes than those with 3′ overhangs of DNA-functionalized nanoparticles and that DNA-functionalized nanoparticles caused a minor biological reaction in HeLa cells, as evidenced by genome-wide expression profiling. Because of their capacity to bind and condense large nucleic acids into nano-sized structures, cationic polymers such as polyethyleneimine are frequently used as transfection agents. This aids the efficient cellular absorption of nucleic acids. Chou et al. demonstrated the construction of DNA-NP superstructures for improved tumour growth and eradication by reducing macrophage sequestration [167]. DNA-NPs were shown to be highly effective in being taken up by virtually all cell types in the absence of transfection agents. Rosi et al. validated for the very first time in 2006 how DNA-NPs transported “antisense” oligonucleotides to eukaryotic cells, together with superior, enhanced, green fluorescence protein (EGFP) knockdown [168]. Giljohann et al. later described the effective transport of siRNA molecules in human cancer cell lines using polyvalent RNA-gold NPs (RNA-Au NPs). The RNA-Au NPs had a longer half-life than free dsRNA, could enter cells without the need for transfection agents, and had strong gene knockdown capabilities in vitro [166]. DNA-NPs were used to target genes such as Bcl2L12, miR-182, ganglioside GM3 synthase, EGFR, and Malat-1. Jensen et al. tested an RNA interference (RNAi)-centred nanotheranostic for oncogene neutralisation in glioblastoma multiforme (GBM). AuNPs were fused with tightly packed and strongly aligned si-RNA duplexes to create DNA-NPs. In glioblastoma multiforme mouse models, the nanoparticles penetrated the blood–brain barrier (BBB) and accumulated all across the tumour mass [169]. The NPs were created to attack the p53 inhibitor directly, an oncoprotein called Bcl2Like12 (Bcl2L12), and effector caspases, which are highly expressed in GBM compared with the healthy brain. Protein levels and endogenous Bcl2L12 mRNA were effectively knocked down, and glioma cells experienced therapy-induced death due to the increase in p53 activity and effector caspase. NPs were later produced to administer miRNA and siRNA to intracranial glioblastoma multiforme tumour locations using a similar strategy [170]. To test their effectiveness in vivo, researchers created a reporter xenograft model that could co-express zn NIR fluorescent protein (iRFP670) and optical reporters for luciferase. The suppression of DNA repair protein O6-methylguanine-DNA-methyltransferase (MGMT; associated with treatment resistance in glioblastoma multiforme) using nanoparticles containing MGMT-targeting siRNA duplexes was quantified using non-invasive optical imaging. A universal injection of nanoparticles into a single tail vein was demonstrated to knock down the MGMT protein in the brain effectively. Furthermore, nanoparticle pharmacokinetics and biodistribution demonstrated fast intra-tumoral retention and absorption, enhancing the anticancer efficacy of temozolomide (TMZ) when given together. Histopathology and blood chemistry tests confirmed that these NPs had no discernible toxicity. Table 11 shows the applications and types of DNA-functionalized NPs

## 6. Persistent Luminescent Nanoparticle (PLNP)-Guided PTT

PLNPs are a type of hollow/mesoporous optical material with a nanocarrier structure suitable for drug administration and a persistent luminescence (PersL) feature that can be employed in treating cancer. They may be created using a variety of emission wavelengths (UV to NIR). They can retain a portion of the excitation energy and then produce photonic emission for an extended period after the excitation is stopped [103]. Because of their variable surface functionality, PLNPs can be employed as nanoplatforms for PersL imaging-guided treatment. Chemodrugs, photothermal agents, genes, or photosensitizers (PSs) can be packed into nanoplatforms. PLNPs are made up of three main components [176]. The host serves as an emitter carrier, and the emission and shape of emitters are determined by the host’s composition and structure [177]. In PLNPs, the emitter has ions such as Eu^2+^, Sm^3+^, Cr^3+^, Mn^2+^, Bi^3+^, and others that are unique, transition metal ions, and main group elements. These emitters determine the luminous wavelength of persistent luminescent nanoparticles [178,179]. The traps are intrinsic flaws in the host or ion doping, influencing the PL duration and intensity [47,180]. PersL is traditionally produced through a solid-state reaction at high temperatures [103], However, sol–gel, template, hydrothermal/solvothermal, and co-synthesis techniques are used in biomedicine. The surface functionalization of PLNPs, such as silicon coating and hydroxylation, which is an exterior modification approach performed by eroding NaOH on the surface of persistent luminescent nanoparticles, is required for future biomedical applications. Moreover, photothermal therapy involves the use of photo-absorbing materials that absorb laser light to generate sufficient heat to kill cancer cells. Photosorber-based PTT has been used in numerous preclinical studies due to its superiority in terms of minimal invasiveness and spatial specificity. PLNPs cannot be used directly in photothermal therapy because of their low extinction coefficient. They incorporate near-infrared (NIR) materials to achieve PersL imaging-guided PTT. The developed nanoplatform possesses significant INR absorption and a good photothermal response, demonstrating effective tumour eradication in vitro and in vivo. Table 12 shows the applications and types of PLNPs.

### Porphyrin-Loaded Nanoparticles

Porphyrins accumulate more in malignant tissues than healthy ones, making them ideal for cancer imaging and treatment. Due to reactive oxygen species (ROS) that are activated by ultrasound or light, they are employed as photosensitizers for cancer (PDT or SDT). They are effective in transporting radioisotopes in radiotherapy because they are effective metal chelators. Porphyrins can be radioisotopically labelled and combined with magnetic resonance imaging agents, which are responsible for multifunctional probes for positron emission tomography (PET) and magnetic resonance imaging [184]. They may be activated with visible light and produce near-infrared or red fluorescence, which can be used for diagnostic fluorescence imaging to assess intracellular localization and therapy efficacy. The most difficult aspect of PDT is getting hydrophobic porphyrins to the targeted locations. Nanoparticles have been reported to naturally aggregate in solid tumours via the enhanced permeation and retention (EPR) effect caused by the combination of leaky vasculature, inadequate increased vascular permeability, and lymphatic drainage [185]. We can generate unique tissue lifetime, targeting, immunological tolerance, hydrophilicity, and other features for porphyrins by attaching them to or encapsulating them into nanoparticles, making them more suited for tissue administration. Entrapping the çhlorin of 2-devinyl-2-(1-hexyloxyethyl)pyropheophorbide into organically modified silica-based nanoparticles resulted in stable monodispersed nanoparticles with higher fluorescence in aqueous solution than the free drug and effective uptake by tumour cells in vitro, according to one study [186]. For near-infrared fluorescence imaging-assisted PDT therapy of gastric cancer tumours in vivo, researchers established a new theranostic program based on chlorin e6 (Ce6)-conjugated carbon dots with remarkable tumour-homing capabilities [187]. The most recent advancements in metallic/metalloid NPs are shown in Table 13, and Table 14 shows the applications and types of porphyrin NPs.

## 7. Limitations and Challenges in Cancer Nanotheranostics

The selection of various polymers and other materials must be based on their profiles of biocompatibility and biodegradability. Additionally, various modifications can be incorporated in order to conceal the toxicity of gold particles and attain the desired properties. For instance, a silica coating on gold particles is also used as a therapeutic material and has been investigated as a carrier for various dyes, imaging mechanisms, and therapeutic agents. Although it has been concluded that the use of biocompatible nanoparticles is more advantageous due to the fact that they are less toxic and other elements can be used to mitigate their effects, this does not preclude the use of toxic nanoparticles. The successful clinical translation of tumour-specific nanoparticle delivery needs to overcome multiple biological constraints and exhibit superior therapeutic efficacy in comparison with the current standard of care [192]. A tumour absorption and tumour visualisation study with anti-EGFR-coated gold nanoparticles of 20 nm in size demonstrated high tumour uptake, while gold nanoparticles of 50 nm in size demonstrated the greatest CT contrast enhancement. The aforementioned study indicated that the size-dependent distribution of theranostic nanomedicines in tumours restricts their use as theranostic agents [193]. Table 15 illustrates the nanoparticle circulation time in cancer therapy. In addition, through Table 16 it was attempted to describe current studies on cancer therapies related to nanotheranostic.

## 8. Conclusions

Nanotechnology has been recognised as a prominent field of study, expanding into various biomedical fields, including therapeutics, imaging, and diagnostics. Due to their versatility and differing morphological characteristics, metallic nanoparticles are an essential field of study. In this review, we describe various metallic nanoparticles and their modified and hybrid versions to understand their role in cancer therapy. Cancer theranostics is a vast field that needs to be investigated, and it shows various challenges and limitations due to the size and biocompatibility of nanoparticles. In all the studies, metallic nanoparticles such as iron and silica nanoparticles were considered more beneficial because of their lesser toxic degradation and their use of less energy to modify themselves as nanoparticles. Despite advancements and promises, nanotheranostic systems need to be significantly improved before they can be used in clinics. The safety, stability, and complexity of nanoparticles must be prioritised when designing nanotheranostics.

## Figures and Tables

**Table 1:** Methods for the synthesis of metallic/metalloid NPs.

Type of NP	Method of Synthesis	References
FeNPs	Co-precipitation, hydrothermal synthesis, microemulsion	[1,10,11,12]
AuNPs	Block co-polymer method	[1,11,12]
ZnNPs	Precipitation, solid-state pyrolysis, wet chemical synthesis	[1,11,12]
AgNPs	Chemical, physical, and biological synthesis	[1,11,12]
CdNPs	Microwave irradiation Photochemical synthesis	[1,10,11,12]

**Table 2:** Different types of Au-NPs used in cancer therapy.

Therapeutic Entity	Type of Au NP	Application
LIN	LIN-AuNPs	Breast cancer
K	K-AuNPs	Breast cancer
PI	PI-AuNPs	Colon and breast cancer
DOX	DOX-PEC-AuNP	Hepatocarcinoma cells
5-FU	AuNP-PEG-5Fu-FA	Cholangiocarcinoma cells
DTX	DTX-HA-cl-AuNP	Anticancer therapy

**Table 3:** Types and applications of ZnO NPs in cancer treatment.

Type of ZnO NP	Application
ZnO-peptide	Colon cancer
Dox-ZnO	Hepatocarcinoma
RGD (Arg-Gly-Asp)-targeted ZnO	Breast cancer
ZnO NPs and Al-ZnO NPs	Lung cancer
DOX-ZnO/PEG nanocomposites	Cervical cancer
PMMA-AA/ZnO NPs and PMMA-PEG/ZnO w	Gastric cancer
HA/ZnO nanocomposites	Acute promyelocytic leukaemia

**Table 4:** Types of AgNPs and their applications in cancer therapy.

Therapeutic Entity	Type of Ag NP	Application	References
Doxorubicin (DOX)	Tat-FeAgNP-Dox	Anti-tumour	[34]
Olax Scanden		Anticancer	[35]
cisplatin (CDDP)	AgNPs/CDDP	Synergistic cellular response	[36]
PEGylated bovine serum albumin AND Indocyanine green	PEG-BSA-AgNP/ICG	Photothermal cancer therapy	[37]
Gallic acid (GA)	GA-AgNPs	Cancer treatment and therapy	[38]

**Table 6:** Types of chalcogenide nanoparticles with their applications in cancer.

Entity	Type of Nanoparticle	Type of Cancer	References
Tellurium chalcogenide nanoparticles	TeNPs	Melanoma	[62,63]
Silver chalcogenides	Ag_2_X	Anticancer	[64]
Copper chalcogenide hybrid nanostructures	Au@Cu_2−*x*_S	Anticancer	[65]
Non-stoichiometric copper chalcogenides	Cu_2−*x*_Se NPs	Anticancer	[65,66]
Selenium chalcogenide nanoparticles	SeNPs	Breast cancer	[65,67]

**Table 7:** Types of hybrid nanoparticles and their applications in cancer therapy.

Entity	Type of NP	Type of Cancer	References
Gold nanoparticles (GNPOPs)-single wall carbon nanotubes (SWCNts)		Breast cancer	[123]
RBC-B16 hybrid membrane camouflaged doxorubicin (DOX)-loaded hollow copper sulphide nanoparticles	DCuS@[RBC-B16] NPs	Melanoma	[124]
Dendrimer-entrapped gold nanoparticles	Au DENPs-FA	Lung cancer	[125]
Polymer lipid hybrid nanoparticles (PLNs) plus doxorubicin (Dox)	Dox-PLNs	Breast cancer	[126]
Hybrid elastin like polypeptide/liposome nanoparticles		Prostate cancer	[127]
Core-shell lipid-polymer hybrid nanoparticles	CSLPHNPs	Prostate cancer	[128]
Sialic acid-modified chitosan-PLGA hybrid nanoparticles	SC-PLGA NPs	Lymphoma	[129]
Genistein-PEGylated silica hybrid nanomaterials	Gen-PEG-SiHNMs	Colon cancer	[130]

**Table 8:** Various types of magnetic nanoparticle and their drug delivery systems and applications in various types of cancer.

Nanoparticle	Type of Cancer	Drug Delivery System	References
Magnetic iron oxide nanoparticles	Breast cancer	siRNA and miRNA co-delivery system	[136]
Doxorubicin-loaded, aptamer-mesoporous silica nanoparticles (MSNs)	Breast cancer	Conjugation of aptamers (targeting agents) and endo/lysosomal escape	[137]
SLNs (solid lipid-based nanoparticles)	Lung cancer	Site-specific drug delivery	[138]
Super-magnetic iron oxide nanoparticles (SPIONs)	Lung cancer	Composite inhalable drug delivery systems	[139]
Liposome, mesoporous silica nanoparticles	T-cell lymphoma	Interleukin 2-diptheria toxin fusion protein (Deniliekin, Diffitox)	[140]

**Table 9:** Types of magnetic nanoparticles and their applications in cancer therapy.

Entity	Type of NPs	Type of Cancer	References
Super magnetic iron oxide nanoparticles	SPIONs	Lung cancer	[145]
Super magnetic iron oxide nanoparticles	MF66	Breast cancer	[146]
Iron oxide NPs with fourth-generation polyamidoamine	G_4_@IOPs	Breast cancer	[147]
Magnetic iron oxide nanoparticles	MIONPs	Prostate cancer	[148]
Magnetic, solid, lipid nanoparticles composed of iron cores with glyceryl trimyristate solid matrix	SLN	Colon cancer	[149]
Doxorubicin with SPIONs	DOX@FASPIONs	Breast cancer	[150]

**Table 10:** Nanotheranostics against types of cancer using magnetic robots.

Entity	Type of NP	Type of Cancer	References
Magnesium-based magneto-fluorescent nanorobots	MFNs	Breast cancer	[153]
Nickel nanorobots	Ni-Ag	Cervical cancer	[154]

**Table 11:** Types of DNA-based nanoparticles with their applications in cancer therapy.

Therapeutic Entity	Type of DNA NP	Application	References
Gold	DNA-Au NPs	Colorectal cancer	[171]
DNA-gated nitrogen-doped carbon quantum dots-loaded hollow mesoporous silica nanoparticles	DNA-gated N-CDs@SiO_2_ NPs	Breast cancer	[172]
Tris amine (HN_3_)	IONP-HN_3_-DNA	Anticancer	[173]
Cu-Au alloy nanostructures coated in Cy5-labeled DNA molecules	Au@Au/Ag NPs	Imaging and PTT of lung cancer	[174]
Gold nanorods	AuNPs with silver and silica shell	Targeted imaging and PTT of ovarian cancer and GBM	[160]
Lanthanum-doped up-conversion nanoparticles with silica shell		Targeted photodynamic therapy for breast cancer	[175]

**Table 12:** Types of PLNPs with their applications in cancer therapy.

Therapeutic Entity	Type of PLNP	Application	References
ZIF8	PLNPs@ZIF-8	Acid-activated tumour imaging and drug release	[181]
PLNP- and ICG-co-loaded mesoporous silica nanoparticles	(PLP+ICG)MISO_2_	Anticancer	[182]
Persistent luminescence-polypyrrole nanocomposites	LPLNP@SPP	Mammary cancer	[183]

**Table 13:** Recent advancements in metallic/metalloid NPs.

Type of NP	Recent Advancement	References
Au NPs	DNA grafting	[158,159,160]
Au NPs, Ag NPs	Polymer coating	[116,117,118]
Fe NPs	Functional silica coating	[105,106,107,108]
Au NPs	Encapsulation of photosensitizers	[16,17,18,19,20]
Au NPs, Ag NPs	Coating with tumour-specific ligands or antibodies	[21,22,32,33]
Fe NPs	Encapsulation of anticancer drugs	[188]
Fe NPs	Aptamer coating	[45,46]

**Table 14:** Types of porphyrin NPs with their applications in cancer therapy.

Therapeutic Entity	Type of Porphyrin NP	Application	References
meso-tetrakis (4-sulphonatophenyl) porphyrin/QCS-SH/gold nanoparticles	TPPS/QCS-SH/AuNPs	Anticancer therapy	[184]
Gelatin	A4por-GNPs	Anticancer therapy	[189]
methoxypolyethyleneglycol-thiol-SPIONs-gold-meso-tetrakis(4-hydroxyphenyl) porphyrin		Breast cancer	[190]
Doxorubicin and meso-tetrakis(4-sulfonatophenyl) porphyrin (TPPS) armoured on gold nanoparticles	DOX@TPPS-AuNPs	Breast cancer	[191]

**Table 15:** Circulation time of nanoparticles in cancer therapy.

Nanoparticle	Circulation Time	References
Gold NPs	More than 24 h after accumulation	[194]
Silver NPs	90 days; in pregnant female mice, 1 to 4 days	[195]
Zinc oxide NPs	24 h after administration	[8]
Iron NPs	24 -36 h after administration	[196]

**Table 16:** Recent literature studies on nanotheranostic cancer therapy.

Title	Publication Year	Remarks	Accession Date	References
Cancer Nanotheranostics: A Nanomedicinal Approach for Cancer Therapy and Diagnosis	2020	In this study, multimodal therapeutic nanoprobes were used in cancer therapy and diagnosis.	24 November 2022	[197]
The Role of Magnetic Nanoparticles in Cancer Nanotheranostics	2020	This study described the role of magnetic nanoparticles as nanotheranostic agents for drug delivery in cancer therapy.	24 November 2022	[198]
Current Trends in Cancer Nanotheranostics: Metallic, Polymeric, and Lipid-Based Systems	2019	The study focused on skin cancer treatment using hybrid nanoparticles.	24 November 2022	[199]
Gold Nanoparticles; Potential Nanotheranostic Agent in Breast Cancer: A Comprehensive Review with Systematic Search Strategy	2020	In this study, gold nanoparticles were used as potential nanothernostic agents to treat breast cancer.	24 November 2022	[200]
A Novel Theranostic Platform: Integration of Magnetomotive and Thermal Ultrasound Imaging With Magnetic Hyperthermia	2021	This study described how magnetic nanoparticles can be used as potential theranostic agents for drug delivery in various temperature ranges.	24 November 2022	[201]
Conjugated-Polymer-Based Nanomaterials for Photothermal Therapy	2020	This study focused on conjugated polymer-based nanomaterials that could be employed as useful photothermal agents for the treatment of numerous diseases.	24 November 2022	[202]
Copper-based nanomaterials for cancer theranostics	2022	The study focused on a copper-based nanomaterial, which can be used as a potential theranostic agent for drug delivery and can also be conjugated with PTT for image-related diagnosis and further treatment.	24 November 2022	[203]

## Data Availability

Not applicable.

## References

[1] Madamsetty V.S., Mukherjee A., Mukherjee S. (2019). Recent Trends of the Bio-Inspired Nanoparticles in Cancer Theranostics. Front. Pharmacol..

[2] Zhu Z., Huang H., Xu Y., Wang M., Lv J., Xu L., Shi C., Xu Y., Yang R., Chen L. (2021). Emergence and Genomics of OXA-232-Producing Klebsiella Pneumoniae in a Hospital in Yancheng, China. J. Glob. Antimicrob. Resist..

[3] Shanbhag P.P., Jog S.V., Chogale M.M., Gaikwad S.S. (2013). Theranostics for Cancer Therapy. Curr. Drug Deliv..

[4] Li J., Gupta S., Li C. (2013). Research Perspectives: Gold Nanoparticles in Cancer Theranostics. Quant. Imaging Med. Surg..

[5] Khan M.S., Vishakante G.D., Siddaramaiah H. (2013). Gold Nanoparticles: A Paradigm Shift in Biomedical Applications. Adv. Colloid Interface Sci..

[6] Ahmed N., Fessi H., Elaissari A. (2012). Theranostic Applications of Nanoparticles in Cancer. Drug Discov. Today.

[7] Kleibert A., Rosellen W., Getzlaff M., Bansmann J. (2011). Structure, Morphology, and Magnetic Properties of Fe Nanoparticles Deposited onto Single-Crystalline Surfaces. Beilstein J. Nanotechnol..

[8] Rasmussen J.W., Martinez E., Louka P., Wingett D.G. (2010). Zinc Oxide Nanoparticles for Selective Destruction of Tumor Cells and Potential for Drug Delivery Applications. Expert Opin. Drug Deliv..

[9] Thomas R., Park I.-K., Jeong Y. (2013). Magnetic Iron Oxide Nanoparticles for Multimodal Imaging and Therapy of Cancer. Int. J. Mol. Sci..

[10] Zhao Y., Zhao Z., Wang Y., Zhou Y., Ma Y., Zuo W. (2020). Single-Cell RNA Expression Profiling of ACE2, the Receptor of SARS-CoV-2. Am. J. Respir. Crit. Care Med..

[11] Dhand C., Dwivedi N., Loh X.J., Jie Ying A.N., Verma N.K., Beuerman R.W., Lakshminarayanan R., Ramakrishna S. (2015). Methods and Strategies for the Synthesis of Diverse Nanoparticles and Their Applications: A Comprehensive Overview. RSC Adv..

[12] Olawale F., Ariatti M., Singh M. (2021). Biogenic Synthesis of Silver-Core Selenium-Shell Nanoparticles Using Ocimum Tenuiflorum L.: Response Surface Methodology-Based Optimization and Biological Activity. Nanomaterials.

[13] Yaqoob A.A., Ahmad H., Parveen T., Ahmad A., Oves M., Ismail I.M.I., Qari H.A., Umar K., Mohamad Ibrahim M.N. (2020). Recent Advances in Metal Decorated Nanomaterials and Their Various Biological Applications: A Review. Front. Chem..

[14] Sharma H., Mishra P.K., Talegaonkar S., Vaidya B. (2015). Metal Nanoparticles: A Theranostic Nanotool against Cancer. Drug Discov. Today.

[15] Li J., Zhang X., Gao F., Yuan Q., Zhang C., Yuan H., Liu Y., Chen L., Han Y., Gao X. (2021). Catalytic Clusterbody for Enhanced Quantitative Protein Immunoblot. Anal. Chem..

[16] Link S., El-Sayed M.A. (2000). Shape and Size Dependence of Radiative, Non-Radiative and Photothermal Properties of Gold Nanocrystals. Int. Rev. Phys. Chem..

[17] Link S., Furube A., Mohamed M.B., Asahi T., Masuhara H., El-Sayed M.A. (2002). Hot Electron Relaxation Dynamics of Gold Nanoparticles Embedded in MgSO _4_ Powder Compared To Solution: The Effect of the Surrounding Medium. J. Phys. Chem. B.

[18] Link S., El-Sayed M.A. (2003). Optical Properties and Ultrafast Dynamics of Metallic Nanocrystals. Annu. Rev. Phys. Chem..

[19] Jain P.K., Lee K.S., El-Sayed I.H., El-Sayed M.A. (2006). Calculated Absorption and Scattering Properties of Gold Nanoparticles of Different Size, Shape, and Composition: Applications in Biological Imaging and Biomedicine. J. Phys. Chem. B.

[20] Riley R.S., Day E.S. (2017). Gold Nanoparticle-mediated Photothermal Therapy: Applications and Opportunities for Multimodal Cancer Treatment. WIREs Nanomed. Nanobiotechnol..

[21] Huang X., Jain P.K., El-Sayed I.H., El-Sayed M.A. (2007). Gold Nanoparticles: Interesting Optical Properties and Recent Applications in Cancer Diagnostics and Therapy. Nanomedicine.

[22] Hwang S., Nam J., Jung S., Song J., Doh H., Kim S. (2014). Gold Nanoparticle-Mediated Photothermal Therapy: Current Status and Future Perspective. Nanomedicine.

[23] Elsayed I., Huang X., Elsayed M. (2006). Selective Laser Photo-Thermal Therapy of Epithelial Carcinoma Using Anti-EGFR Antibody Conjugated Gold Nanoparticles. Cancer Lett..

[24] Ren Y., Yan Y., Qi H. (2022). Photothermal Conversion and Transfer in Photothermal Therapy: From Macroscale to Nanoscale. Adv. Colloid Interface Sci..

[25] Hleb E.Y., Hafner J.H., Myers J.N., Hanna E.Y., Rostro B.C., Zhdanok S.A., Lapotko D.O. (2008). LANTCET: Elimination of Solid Tumor Cells with Photothermal Bubbles Generated around Clusters of Gold Nanoparticles. Nanomedicine.

[26] Wang Z., Chen Z., Liu Z., Shi P., Dong K., Ju E., Ren J., Qu X. (2014). A Multi-Stimuli Responsive Gold Nanocage–Hyaluronic Platform for Targeted Photothermal and Chemotherapy. Biomaterials.

[27] Gao L., Liu R., Gao F., Wang Y., Jiang X., Gao X. (2014). Plasmon-Mediated Generation of Reactive Oxygen Species from Near-Infrared Light Excited Gold Nanocages for Photodynamic Therapy in Vitro. ACS Nano.

[28] Gao L., Fei J., Zhao J., Li H., Cui Y., Li J. (2012). Hypocrellin-Loaded Gold Nanocages with High Two-Photon Efficiency for Photothermal/Photodynamic Cancer Therapy *in Vitro*. ACS Nano.

[29] Gao L., Liu M., Ma G., Wang Y., Zhao L., Yuan Q., Gao F., Liu R., Zhai J., Chai Z. (2015). Peptide-Conjugated Gold Nanoprobe: Intrinsic Nanozyme-Linked Immunsorbant Assay of Integrin Expression Level on Cell Membrane. ACS Nano.

[30] Hanley C., Layne J., Punnoose A., Reddy K.M., Coombs I., Coombs A., Feris K., Wingett D. (2008). Preferential Killing of Cancer Cells and Activated Human T Cells Using ZnO Nanoparticles. Nanotechnology.

[31] Sharma S.K., Pujari P.K., Sudarshan K., Dutta D., Mahapatra M., Godbole S.V., Jayakumar O.D., Tyagi A.K. (2009). Positron Annihilation Studies in ZnO Nanoparticles. Solid State Commun..

[32] Sironmani A., Daniel K., Kapetanovi I. (2011). Silver Nanoparticles – Universal Multifunctional Nanoparticles for Bio Sensing, Imaging for Diagnostics and Targeted Drug Delivery for Therapeutic Applications. Drug Discovery and Development-Present and Future.

[33] Mohammadzadeh R. (2012). Hypothesis: Silver Nanoparticles as an Adjuvant for Cancertherapy. Adv. Pharm. Bull..

[34] Gregg V., Milligan L.P. (1980). Inhibition of Na+, K+-ATPase of Intact Mouse Soleus Muscle by Mg++. Biochem. Biophys. Res. Commun..

[35] Ovais M., Khalil A.T., Raza A., Khan M.A., Ahmad I., Islam N.U., Saravanan M., Ubaid M.F., Ali M., Shinwari Z.K. (2016). Green Synthesis of Silver Nanoparticles via Plant Extracts: Beginning a New Era in Cancer Theranostics. Nanomedicine.

[36] Rank Miranda R., Pereira da Fonseca M., Korzeniowska B., Skytte L., Lund Rasmussen K., Kjeldsen F. (2020). Elucidating the Cellular Response of Silver Nanoparticles as a Potential Combinatorial Agent for Cisplatin Chemotherapy. J. Nanobiotechnol..

[37] Park T., Lee S., Amatya R., Cheong H., Moon C., Kwak H.D., Min K.A., Shin M.C. (2020). ICG-Loaded PEGylated BSA-Silver Nanoparticles for Effective Photothermal Cancer Therapy. Int. J. Nanomed..

[38] Nemčeková K., Svitková V., Sochr J., Gemeiner P., Labuda J. (2022). Gallic Acid-Coated Silver Nanoparticles as Perspective Drug Nanocarriers: Bioanalytical Study. Anal. Bioanal. Chem..

[39] Cole A.J., Yang V.C., David A.E. (2011). Cancer Theranostics: The Rise of Targeted Magnetic Nanoparticles. Trends Biotechnol..

[40] Xie J., Lee S., Chen X. (2010). Nanoparticle-Based Theranostic Agents. Adv. Drug Deliv. Rev..

[41] Singh A., Sahoo S.K. (2014). Magnetic Nanoparticles: A Novel Platform for Cancer Theranostics. Drug Discov. Today.

[42] Sun C., Fang C., Stephen Z., Veiseh O., Hansen S., Lee D., Ellenbogen R.G., Olson J., Zhang M. (2008). Tumor-Targeted Drug Delivery and MRI Contrast Enhancement by Chlorotoxin-Conjugated Iron Oxide Nanoparticles. Nanomedicine.

[43] Shahbazi-Gahrouei D., Abdolahi M. (2013). Detection of MUC1-Expressing Ovarian Cancer by C595 Monoclonal Antibody-Conjugated SPIONs Using MR Imaging. Sci. World J..

[44] Ma X., Gong A., Chen B., Zheng J., Chen T., Shen Z., Wu A. (2015). Exploring a New SPION-Based MRI Contrast Agent with Excellent Water-Dispersibility, High Specificity to Cancer Cells and Strong MR Imaging Efficacy. Colloids Surf. B Biointerfaces.

[45] Pilapong C., Sitthichai S., Thongtem S., Thongtem T. (2014). Smart Magnetic Nanoparticle-Aptamer Probe for Targeted Imaging and Treatment of Hepatocellular Carcinoma. Int. J. Pharm..

[46] Lin G., Zhu W., Yang L., Wu J., Lin B., Xu Y., Cheng Z., Xia C., Gong Q., Song B. (2014). Delivery of SiRNA by MRI-Visible Nanovehicles to Overcome Drug Resistance in MCF-7/ADR Human Breast Cancer Cells. Biomaterials.

[47] Li Y., Zhou S., Li Y., Sharafudeen K., Ma Z., Dong G., Peng M., Qiu J. (2014). Long Persistent and Photo-Stimulated Luminescence in Cr3+-Doped Zn–Ga–Sn–O Phosphors for Deep and Reproducible Tissue Imaging. J. Mater. Chem. C.

[48] Rajan A., Sahu N.K. (2020). Review on Magnetic Nanoparticle-Mediated Hyperthermia for Cancer Therapy. J. Nanopart. Res..

[49] Zhao Y., Zhao X., Cheng Y., Guo X., Yuan W. (2018). Iron Oxide Nanoparticles-Based Vaccine Delivery for Cancer Treatment. Mol. Pharm..

[50] Chen Q., Ma X., Xie L., Chen W., Xu Z., Song E., Zhu X., Song Y. (2021). Iron-Based Nanoparticles for MR Imaging-Guided Ferroptosis in Combination with Photodynamic Therapy to Enhance Cancer Treatment. Nanoscale.

[51] Li J., Li L., Lv Y., Zou H., Wei Y., Nie F., Duan W., Sedike M., Xiao L., Wang M. (2020). The Construction of the Novel Magnetic Prodrug Fe3O4@DOX and Its Antagonistic Effects on Hepatocarcinoma with Low Toxicity. RSC Adv..

[52] Huang C.-H., Chuang T.-J., Ke C.-J., Yao C.-H. (2020). Doxorubicin–Gelatin/Fe_3_O_4_–Alginate Dual-Layer Magnetic Nanoparticles as Targeted Anticancer Drug Delivery Vehicles. Polymers.

[53] Fischer W. (2001). A Second Note on the Term “Chalcogen". J. Chem. Educ..

[54] Shevchenko N., Steinhart M., Tomšík E. (2019). Single-Step Preparation of Mono-Dispersed Sulfur Nanoparticles for Detention of Copper. J. Nanopart. Res..

[55] Tripathi R.M., Rao R.P., Tsuzuki T. (2018). Green Synthesis of Sulfur Nanoparticles and Evaluation of Their Catalytic Detoxification of Hexavalent Chromium in Water. RSC Adv..

[56] Shankar C., Basu S., Lal B., Shanmugam S., Vasudevan K., Mathur P., Ramaiah S., Anbarasu A., Veeraraghavan B. (2021). Aerobactin Seems To Be a Promising Marker Compared With Unstable RmpA2 for the Identification of Hypervirulent Carbapenem-Resistant Klebsiella Pneumoniae: In Silico and In Vitro Evidence. Front. Cell. Infect. Microbiol..

[57] Rai M., Ingle A.P., Paralikar P. (2016). Sulfur and Sulfur Nanoparticles as Potential Antimicrobials: From Traditional Medicine to Nanomedicine. Expert Rev. Anti-Infect. Ther..

[58] Castro L., Li J., González F., Muñoz J.A., Blázquez M.L. (2020). Green Synthesis of Tellurium Nanoparticles by Tellurate and Tellurite Reduction Using Aeromonas Hydrophila under Different Aeration Conditions. Hydrometallurgy.

[59] Xiao M., Yang L.U.S. (2014). Binary and Ternary Metal Chalcogenide Materials and Method of Making and Using Same. U.S. Patent.

[60] Ahmed A.J.A., Alaa H.A.A. (2016). Virulence Factors and Antibiotic Susceptibility Patterns of Multidrug Resistance Klebsiella Pneumoniae Isolated from Different Clinical Infections. Afr. J. Microbiol. Res..

[61] Ingale A.G. (2013). Biogenic Synthesis of Nanoparticles and Potential Applications: An Eco- Friendly Approach. J. Nanomed. Nanotechnol..

[62] Medina Cruz D., Tien-Street W., Zhang B., Huang X., Vernet Crua A., Nieto-Argüello A., Cholula-Díaz J.L., Martínez L., Huttel Y., González M.U. (2019). Citric Juice-Mediated Synthesis of Tellurium Nanoparticles with Antimicrobial and Anticancer Properties. Green Chem..

[63] Olawale F., Oladimeji O., Ariatti M., Singh M. (2022). Emerging Roles of Green-Synthesized Chalcogen and Chalcogenide Nanoparticles in Cancer Theranostics. J. Nanotechnol..

[64] Nieves L.M., Mossburg K., Hsu J.C., Maidment A.D.A., Cormode D.P. (2021). Silver Chalcogenide Nanoparticles: A Review of Their Biomedical Applications. Nanoscale.

[65] Yan C., Tian Q., Yang S. (2017). Recent Advances in the Rational Design of Copper Chalcogenide to Enhance the Photothermal Conversion Efficiency for the Photothermal Ablation of Cancer Cells. RSC Adv..

[66] Liu K., Liu K., Liu J., Ren Q., Zhao Z., Wu X., Li D., Yuan F., Ye K., Li B. (2020). Copper Chalcogenide Materials as Photothermal Agents for Cancer Treatment. Nanoscale.

[67] Ramamurthy C., Sampath K.S., Arunkumar P., Kumar M.S., Sujatha V., Premkumar K., Thirunavukkarasu C. (2013). Green Synthesis and Characterization of Selenium Nanoparticles and Its Augmented Cytotoxicity with Doxorubicin on Cancer Cells. Bioprocess. Biosyst. Eng..

[68] Maiyo F., Singh M. (2017). Selenium Nanoparticles: Potential in Cancer Gene and Drug Delivery. Nanomedicine.

[69] Torres S.K., Campos V.L., León C.G., Rodríguez-Llamazares S.M., Rojas S.M., González M., Smith C., Mondaca M.A. (2012). Biosynthesis of Selenium Nanoparticles by Pantoea Agglomerans and Their Antioxidant Activity. J. Nanopart. Res..

[70] Sholkamy E., Ahmad M., Manal Yaser M., Ali A., Mehanni M. (2015). Anticancer Activity of Biostabilized Selenium Nanorods Synthesized by Streptomyces Bikiniensis Strain Ess_amA-1. Int. J. Nanomed..

[71] Bao P., Chen S.-C., Xiao K.-Q. (2015). Dynamic Equilibrium of Endogenous Selenium Nanoparticles in Selenite-Exposed Cancer Cells: A Deep Insight into the Interaction between Endogenous SeNPs and Proteins. Mol. BioSyst..

[72] Ba L.A., Döring M., Jamier V., Jacob C. (2010). Tellurium: An Element with Great Biological Potency and Potential. Org. Biomol. Chem..

[73] Danhier F., Feron O., Préat V. (2010). To Exploit the Tumor Microenvironment: Passive and Active Tumor Targeting of Nanocarriers for Anti-Cancer Drug Delivery. J. Control Release.

[74] DeLeon E.R., Gao Y., Huang E., Arif M., Arora N., Divietro A., Patel S., Olson K.R. (2016). A Case of Mistaken Identity: Are Reactive Oxygen Species Actually Reactive Sulfide Species?. Am. J. Physiol.-Regul. Integr. Comp. Physiol..

[75] Tang S.-M., Deng X.-T., Zhou J., Li Q.-P., Ge X.-X., Miao L. (2020). Pharmacological Basis and New Insights of Quercetin Action in Respect to Its Anti-Cancer Effects. Biomed. Pharmacother..

[76] Huang C., Wang Y., Li X., Ren L., Zhao J., Hu Y., Zhang L., Fan G., Xu J., Gu X. (2020). Clinical Features of Patients Infected with 2019 Novel Coronavirus in Wuhan, China. Lancet.

[77] Galeone C., Pelucchi C., Levi F., Negri E., Franceschi S., Talamini R., Giacosa A., La Vecchia C. (2006). Onion and Garlic Use and Human Cancer. Am. J. Clin. Nutr..

[78] Mates J.M. (2012). Sulphur-Containing Non Enzymatic Antioxidants Therapeutic Tools against Cancer. Front. Biosci..

[79] Zahran F., Hammadi M., Al-dulaimi M., Sebaiy M. (2018). Potential Role of Sulfur Nanoparticles as Antitumor and Antioxidant in Mice. Pharm. Lett..

[80] Kim J.-H., Jang H.-J., Cho W.-Y., Yeon S.-J., Lee C.-H. (2020). In Vitro Antioxidant Actions of Sulfur-Containing Amino Acids. Arab. J. Chem..

[81] Levine R.L., Mosoni L., Berlett B.S., Stadtman E.R. (1996). Methionine Residues as Endogenous Antioxidants in Proteins. Proc. Natl. Acad. Sci. USA.

[82] Lee J., Lee H.-J., Park J.-D., Lee S.-K., Lee S.-I., Lim H.-D., Lee Y.-M., Yun Y.-G., Jeon B.-H., Ree I.-S. (2008). Anti-Cancer Activity of Highly Purified Sulfur in Immortalized and Malignant Human Oral Keratinocytes. Toxicol. Vitr..

[83] Cho S.J., Maysinger D., Jain M., Röder B., Hackbarth S., Winnik F.M. (2007). Long-Term Exposure to CdTe Quantum Dots Causes Functional Impairments in Live Cells. Langmuir.

[84] Dailianis S., Piperakis S.M., Kaloyianni M. (2005). Cadmium Effects on ROS Production and DNA Damage via Adrenergic Receptors Stimulation: Role of Na^+^/H^+^ Exchanger and PKC. Free Radic. Res..

[85] Hoshino A., Fujioka K., Oku T., Suga M., Sasaki Y.F., Ohta T., Yasuhara M., Suzuki K., Yamamoto K. (2004). Physicochemical Properties and Cellular Toxicity of Nanocrystal Quantum Dots Depend on Their Surface Modification. Nano Lett..

[86] Lai L., Jin J.-C., Xu Z.-Q., Mei P., Jiang F.-L., Liu Y. (2015). Necrotic Cell Death Induced by the Protein-Mediated Intercellular Uptake of CdTe Quantum Dots. Chemosphere.

[87] Kaviyarasu K., Kanimozhi K., Matinise N., Maria Magdalane C., Mola G.T., Kennedy J., Maaza M. (2017). Antiproliferative Effects on Human Lung Cell Lines A549 Activity of Cadmium Selenide Nanoparticles Extracted from Cytotoxic Effects: Investigation of Bio-Electronic Application. Mater. Sci. Eng. C.

[88] Viana O.S., Ribeiro M.S., Fontes A., Santos B.S., Batinić-Haberle I., Rebouças J.S., Spasojević I. (2016). Quantum Dots in Photodynamic Therapy. Redox-Active Therapeutics.

[89] Nakamura M., Ishimura K. (2008). One-Pot Synthesis and Characterization of Three Kinds of Thiol−Organosilica Nanoparticles. Langmuir.

[90] Nakamura M., Ishimura K. (2007). Synthesis and Characterization of Organosilica Nanoparticles Prepared from 3-Mercaptopropyltrimethoxysilane as the Single Silica Source. J. Phys. Chem. C.

[91] Vogel R., Surawski P.P.T., Littleton B.N., Miller C.R., Lawrie G.A., Battersby B.J., Trau M. (2007). Fluorescent Organosilica Micro- and Nanoparticles with Controllable Size. J. Colloid Interface Sci..

[92] Nakamura M., Ishimura K. (2008). Size-Controlled, One-Pot Synthesis, Characterization, and Biological Applications of Epoxy-Organosilica Particles Possessing Positive Zeta Potential. Langmuir.

[93] Herr J.K., Smith J.E., Medley C.D., Shangguan D., Tan W. (2006). Aptamer-Conjugated Nanoparticles for Selective Collection and Detection of Cancer Cells. Anal. Chem..

[94] Medley C.D., Bamrungsap S., Tan W., Smith J.E. (2011). Aptamer-Conjugated Nanoparticles for Cancer Cell Detection. Anal. Chem..

[95] Cai L., Chen Z.-Z., Chen M.-Y., Tang H.-W., Pang D.-W. (2013). MUC-1 Aptamer-Conjugated Dye-Doped Silica Nanoparticles for MCF-7 Cells Detection. Biomaterials.

[96] Kumar R., Roy I., Ohulchanskyy T.Y., Goswami L.N., Bonoiu A.C., Bergey E.J., Tramposch K.M., Maitra A., Prasad P.N. (2008). Covalently Dye-Linked, Surface-Controlled, and Bioconjugated Organically Modified Silica Nanoparticles as Targeted Probes for Optical Imaging. ACS Nano.

[97] Couleaud P., Morosini V., Frochot C., Richeter S., Raehm L., Durand J.-O. (2010). Silica-Based Nanoparticles for Photodynamic Therapy Applications. Nanoscale.

[98] Simon V., Devaux C., Darmon A., Donnet T., ThiÃ©not E., Germain M., Honnorat J., Duval A., Pottier A., Borghi E. (2010). Pp IX Silica Nanoparticles Demonstrate Differential Interactions with In Vitro Tumor Cell Lines and In Vivo Mouse Models of Human Cancers. Photochem. Photobiol..

[99] Li Z., Wang J., Chen J., Lei W., Wang X., Zhang B. (2010). Hypocrellin B Doped and PH-Responsive Silica Nanoparticles for Photodynamic Therapy. Sci. China Chem..

[100] Sandler S.E., Fellows B., Mefford O.T. (2019). Best Practices for Characterization of Magnetic Nanoparticles for Biomedical Applications. Anal. Chem..

[101] Dinali R., Ebrahiminezhad A., Manley-Harris M., Ghasemi Y., Berenjian A. (2017). Iron Oxide Nanoparticles in Modern Microbiology and Biotechnology. Crit. Rev. Microbiol..

[102] Shevtsov M.A., Nikolaev B.P., Ryzhov V.A., Yakovleva L.Y., Dobrodumov A.V., Marchenko Y.Y., Margulis B.A., Pitkin E., Mikhrina A.L., Guzhova I.V. (2016). Detection of Experimental Myocardium Infarction in Rats by MRI Using Heat Shock Protein 70 Conjugated Superparamagnetic Iron Oxide Nanoparticle. Nanomed. Nanotechnol. Biol. Med..

[103] Li Y., Gecevicius M., Qiu J. (2016). Long Persistent Phosphors—from Fundamentals to Applications. Chem. Soc. Rev..

[104] Moliner-Martínez Y., Ribera A., Coronado E., Campíns-Falcó P. (2011). Preconcentration of Emerging Contaminants in Environmental Water Samples by Using Silica Supported Fe3O4 Magnetic Nanoparticles for Improving Mass Detection in Capillary Liquid Chromatography. J. Chromatogr. A.

[105] Ryu J., Lee K., Joe C., Joo J., Lee N., Yoo H.-S. (2018). Patient With Unresectable Cholangiocarcinoma Treated With Radiofrequency Hyperthermia in Combination With Chemotherapy: A Case Report. Integr. Cancer.

[106] Jose J., Kumar R., Harilal S., Mathew G.E., Parambi D.G.T., Prabhu A., Uddin S., Aleya L., Kim H., Mathew B. (2020). Magnetic Nanoparticles for Hyperthermia in Cancer Treatment: An Emerging Tool. Environ. Sci. Pollut. Res..

[107] Czugala M., Mykhaylyk O., Böhler P., Onderka J., Stork B., Wesselborg S., Kruse F.E., Plank C., Singer B.B., Fuchsluger T.A. (2016). Efficient and Safe Gene Delivery to Human Corneal Endothelium Using Magnetic Nanoparticles. Nanomedicine.

[108] Blanco E., Shen H., Ferrari M. (2015). Principles of Nanoparticle Design for Overcoming Biological Barriers to Drug Delivery. Nat. Biotechnol..

[109] Liu J., Li S., Liu J., Liang B., Wang X., Wang H., Li W., Tong Q., Yi J., Zhao L. (2020). Longitudinal Characteristics of Lymphocyte Responses and Cytokine Profiles in the Peripheral Blood of SARS-CoV-2 Infected Patients. eBioMedicine.

[110] Kim D.-H., Rozhkova E.A., Ulasov I.V., Bader S.D., Rajh T., Lesniak M.S., Novosad V. (2010). Biofunctionalized Magnetic-Vortex Microdiscs for Targeted Cancer-Cell Destruction. Nat. Mater..

[111] Seifert G., Budach V., Keilholz U., Wust P., Eggert A., Ghadjar P. (2016). Regional Hyperthermia Combined with Chemotherapy in Paediatric, Adolescent and Young Adult Patients: Current and Future Perspectives. Radiat. Oncol..

[112] Hou H., Wang C., Nan K., Freeman W.R., Sailor M.J., Cheng L. (2016). Controlled Release of Dexamethasone From an Intravitreal Delivery System Using Porous Silicon Dioxide. Investig. Ophthalmol. Vis. Sci..

[113] Diksha, Roy I., Soloviev M. (2012). Synthesis, Surface Modification, Characterization, and Biomedical In Vitro Applications of Organically Modified Silica (ORMOSIL) Nanoparticles. Nanoparticles in Biology and Medicine.

[114] Alonso J., Khurshid H., Devkota J., Nemati Z., Khadka N.K., Srikanth H., Pan J., Phan M.-H. (2016). Superparamagnetic Nanoparticles Encapsulated in Lipid Vesicles for Advanced Magnetic Hyperthermia and Biodetection. J. Appl. Phys..

[115] Albini M., Salvi M., Altamura E., Dinarelli S., Di Donato L., Lucibello A., Mavelli F., Molinari F., Morbiducci U., Ramundo-Orlando A. (2019). Movement of Giant Lipid Vesicles Induced by Millimeter Wave Radiation Change When They Contain Magnetic Nanoparticles. Drug Deliv. Transl. Res..

[116] Zhang S., Yang G., Ye Q., Wu Q., Zhang J., Huang Y. (2018). Phenotypic and Genotypic Characterization of Klebsiella Pneumoniae Isolated From Retail Foods in China. Front. Microbiol..

[117] Zhang J., Misra R.D.K. (2007). Magnetic Drug-Targeting Carrier Encapsulated with Thermosensitive Smart Polymer: Core–Shell Nanoparticle Carrier and Drug Release Response. Acta Biomater..

[118] Chowdhuri A.R., Singh T., Ghosh S.K., Sahu S.K. (2016). Carbon Dots Embedded Magnetic Nanoparticles @Chitosan @Metal Organic Framework as a Nanoprobe for PH Sensitive Targeted Anticancer Drug Delivery. ACS Appl. Mater. Interfaces.

[119] Kandasamy G., Maity D. (2015). Recent Advances in Superparamagnetic Iron Oxide Nanoparticles (SPIONs) for in Vitro and in Vivo Cancer Nanotheranostics. Int. J. Pharm..

[120] Lyer S., Singh R., Tietze R., Alexiou C. (2015). Magnetic Nanoparticles for Magnetic Drug Targeting. Biomed. Eng./Biomed. Tech..

[121] Talluri S., Malla R.R. (2020). Superparamagnetic Iron Oxide Nanoparticles (SPIONs) for Diagnosis and Treatment of Breast, Ovarian and Cervical Cancers. Curr. Drug Metab..

[122] Chan J.M.S., Cheung M.S.H., Gibbs R.G.J., Bhakoo K.K. (2017). MRI Detection of Endothelial Cell Inflammation Using Targeted Superparamagnetic Particles of Iron Oxide (SPIO). Clin. Transl. Med..

[123] Beqa L., Fan Z., Singh A.K., Senapati D., Ray P.C. (2011). Gold Nano-Popcorn Attached SWCNT Hybrid Nanomaterial for Targeted Diagnosis and Photothermal Therapy of Human Breast Cancer Cells. ACS Appl. Mater. Interfaces.

[124] Wang D., Dong H., Li M., Cao Y., Yang F., Zhang K., Dai W., Wang C., Zhang X. (2018). Erythrocyte–Cancer Hybrid Membrane Camouflaged Hollow Copper Sulfide Nanoparticles for Prolonged Circulation Life and Homotypic-Targeting Photothermal/Chemotherapy of Melanoma. ACS Nano.

[125] Mottaghitalab F., Farokhi M., Fatahi Y., Atyabi F., Dinarvand R. (2019). New Insights into Designing Hybrid Nanoparticles for Lung Cancer: Diagnosis and Treatment. J. Control Release.

[126] Wong J., Prout J., Seifalian A. (2017). Magnetic Nanoparticles: New Perspectives in Drug Delivery. Curr. Pharm. Des..

[127] Zhang W., Song Y., Eldi P., Guo X., Hayball J., Garg S., Albrecht H. (2018). Targeting Prostate Cancer Cells with Hybrid Elastin-like Polypeptide/Liposome Nanoparticles. Int. J. Nanomed..

[128] Wang Q., Alshaker H., Böhler T., Srivats S., Chao Y., Cooper C., Pchejetski D. (2017). Core Shell Lipid-Polymer Hybrid Nanoparticles with Combined Docetaxel and Molecular Targeted Therapy for the Treatment of Metastatic Prostate Cancer. Sci. Rep..

[129] Zhang H., Zhao J., Gu X., Wen Y. (2019). Targeted Treatment of CD22-Positive Non-Hodgkin’s Lymphoma with Sialic Acid–Modified Chitosan-PLGA Hybrid Nanoparticles. J. Nanopart. Res..

[130] Pool H., Campos-Vega R., Herrera-Hernández M.G., García-Solis P., García-Gasca T., Sánchez I.C., Luna-Bárcenas G., Vergara-Castañeda H. (2018). Development of Genistein-PEGylated Silica Hybrid Nanomaterials with Enhanced Antioxidant and Antiproliferative Properties on HT29 Human Colon Cancer Cells. Am. J. Transl. Res..

[131] Xiong F., Huang S., Gu N. (2018). Magnetic Nanoparticles: Recent Developments in Drug Delivery System. Drug Dev. Ind. Pharm..

[132] Manshadi M.K.D., Saadat M., Mohammadi M., Shamsi M., Dejam M., Kamali R., Sanati-Nezhad A. (2018). Delivery of Magnetic Micro/Nanoparticles and Magnetic-Based Drug/Cargo into Arterial Flow for Targeted Therapy. Drug Deliv..

[133] Hervault A., Thanh N.T.K. (2014). Magnetic Nanoparticle-Based Therapeutic Agents for Thermo-Chemotherapy Treatment of Cancer. Nanoscale.

[134] Huang Y., Mao K., Zhang B., Zhao Y. (2017). Superparamagnetic Iron Oxide Nanoparticles Conjugated with Folic Acid for Dual Target-Specific Drug Delivery and MRI in Cancer Theranostics. Mater. Sci. Eng. C.

[135] Yang Y., Guo Q., Peng J., Su J., Lu X., Zhao Y., Qian Z. (2016). Doxorubicin-Conjugated Heparin-Coated Superparamagnetic Iron Oxide Nanoparticles for Combined Anticancer Drug Delivery and Magnetic Resonance Imaging. J. Biomed. Nanotechnol..

[136] Liyanage P.Y., Hettiarachchi S.D., Zhou Y., Ouhtit A., Seven E.S., Oztan C.Y., Celik E., Leblanc R.M. (2019). Nanoparticle-Mediated Targeted Drug Delivery for Breast Cancer Treatment. Biochim. Biophys. Acta BBA-Rev. Cancer.

[137] Lohiya G., Katti D.S. (2022). Carboxylated Chitosan-Mediated Improved Efficacy of Mesoporous Silica Nanoparticle-Based Targeted Drug Delivery System for Breast Cancer Therapy. Carbohydr. Polym..

[138] Abdelaziz A.M., Salem S.S., Khalil A.M.A., El-Wakil D.A., Fouda H.M., Hashem A.H. (2022). Potential of Biosynthesized Zinc Oxide Nanoparticles to Control Fusarium Wilt Disease in Eggplant (*Solanum melongena*) and Promote Plant Growth. BioMetals.

[139] Reczyńska K., Marszałek M., Zarzycki A., Reczyński W., Kornaus K., Pamuła E., Chrzanowski W. (2020). Superparamagnetic Iron Oxide Nanoparticles Modified with Silica Layers as Potential Agents for Lung Cancer Treatment. Nanomaterials.

[140] Dianzani C., Zara G.P., Maina G., Pettazzoni P., Pizzimenti S., Rossi F., Gigliotti C.L., Ciamporcero E.S., Daga M., Barrera G. (2014). Drug Delivery Nanoparticles in Skin Cancers. BioMed Res. Int..

[141] Moise S., Byrne J.M., El Haj A.J., Telling N.D. (2018). The Potential of Magnetic Hyperthermia for Triggering the Differentiation of Cancer Cells. Nanoscale.

[142] Salunkhe A.B., Khot V.M., Pawar S.H. (2014). Magnetic Hyperthermia with Magnetic Nanoparticles: A Status Review. Curr. Top. Med. Chem..

[143] Minbashi M., Kordbacheh A.A., Ghobadi A., Tuchin V.V. (2020). Optimization of Power Used in Liver Cancer Microwave Therapy by Injection of Magnetic Nanoparticles (MNPs). Comput. Biol. Med..

[144] Bucci O.M., Bellizzi G., Costanzo S., Crocco L., Di Massa G., Scapaticci R. (2021). Experimental Characterization of Spurious Signals in Magnetic Nanoparticles Enhanced Microwave Imaging of Cancer. Sensors.

[145] Sadhukha T., Wiedmann T.S., Panyam J. (2013). Inhalable Magnetic Nanoparticles for Targeted Hyperthermia in Lung Cancer Therapy. Biomaterials.

[146] Kossatz S., Grandke J., Couleaud P., Latorre A., Aires A., Crosbie-Staunton K., Ludwig R., Dähring H., Ettelt V., Lazaro-Carrillo A. (2015). Efficient Treatment of Breast Cancer Xenografts with Multifunctionalized Iron Oxide Nanoparticles Combining Magnetic Hyperthermia and Anti-Cancer Drug Delivery. Breast Cancer Res..

[147] Salimi M., Sarkar S., Hashemi M., Saber R. (2020). Treatment of Breast Cancer-Bearing BALB/c Mice with Magnetic Hyperthermia Using Dendrimer Functionalized Iron-Oxide Nanoparticles. Nanomaterials.

[148] Attaluri A., Kandala S.K., Wabler M., Zhou H., Cornejo C., Armour M., Hedayati M., Zhang Y., DeWeese T.L., Herman C. (2015). Magnetic Nanoparticle Hyperthermia Enhances Radiation Therapy: A Study in Mouse Models of Human Prostate Cancer. Int. J. Hyperth..

[149] Muñoz de Escalona M., Sáez-Fernández E., Prados J.C., Melguizo C., Arias J.L. (2016). Magnetic Solid Lipid Nanoparticles in Hyperthermia against Colon Cancer. Int. J. Pharm..

[150] Li X., Li W., Wang M., Liao Z. (2021). Magnetic Nanoparticles for Cancer Theranostics: Advances and Prospects. J. Control. Release.

[151] Nikitin M.P., Shipunova V.O., Deyev S.M., Nikitin P.I. (2014). Biocomputing Based on Particle Disassembly. Nat. Nanotechnol..

[152] Li S., Jiang Q., Ding B., Nie G. (2019). Anticancer Activities of Tumor-Killing Nanorobots. Trends Biotechnol..

[153] Wavhale R.D., Dhobale K.D., Rahane C.S., Chate G.P., Tawade B.V., Patil Y.N., Gawade S.S., Banerjee S.S. (2021). Water-Powered Self-Propelled Magnetic Nanobot for Rapid and Highly Efficient Capture of Circulating Tumor Cells. Commun. Chem..

[154] Hu M., Ge X., Chen X., Mao W., Qian X., Yuan W.-E. (2020). Micro/Nanorobot: A Promising Targeted Drug Delivery System. Pharmaceutics.

[155] Jin R., Wu G., Li Z., Mirkin C.A., Schatz G.C. (2003). What Controls the Melting Properties of DNA-Linked Gold Nanoparticle Assemblies?. J. Am. Chem. Soc..

[156] Jones M.R., Seeman N.C., Mirkin C.A. (2015). Programmable Materials and the Nature of the DNA Bond. Science.

[157] Mirkin C.A., Letsinger R.L., Mucic R.C., Storhoff J.J. (1996). A DNA-Based Method for Rationally Assembling Nanoparticles into Macroscopic Materials. Nature.

[158] Zhang Y., Lu F., Yager K.G., van der Lelie D., Gang O. (2013). A General Strategy for the DNA-Mediated Self-Assembly of Functional Nanoparticles into Heterogeneous Systems. Nat. Nanotechnol..

[159] Cutler J.I., Zhang K., Zheng D., Auyeung E., Prigodich A.E., Mirkin C.A. (2011). Polyvalent Nucleic Acid Nanostructures. J. Am. Chem. Soc..

[160] Pal S., Ray A., Andreou C., Zhou Y., Rakshit T., Wlodarczyk M., Maeda M., Toledo-Crow R., Berisha N., Yang J. (2019). DNA-Enabled Rational Design of Fluorescence-Raman Bimodal Nanoprobes for Cancer Imaging and Therapy. Nat. Commun..

[161] Kahn J.S., Freage L., Enkin N., Garcia M.A.A., Willner I. (2017). Stimuli-Responsive DNA-Functionalized Metal-Organic Frameworks (MOFs). Adv. Mater..

[162] Li L.-L., Wu P., Hwang K., Lu Y. (2013). An Exceptionally Simple Strategy for DNA-Functionalized Up-Conversion Nanoparticles as Biocompatible Agents for Nanoassembly, DNA Delivery, and Imaging. J. Am. Chem. Soc..

[163] Liu B., Zheng D., Jin Q., Chen L., Yang J. (2019). VFDB 2019: A Comparative Pathogenomic Platform with an Interactive Web Interface. Nucleic Acids Res..

[164] Li Z., Zhang Y., Fullhart P., Mirkin C.A. (2004). Reversible and Chemically Programmable Micelle Assembly with DNA Block-Copolymer Amphiphiles. Nano Lett..

[165] Kuzyk A., Jungmann R., Acuna G.P., Liu N. (2018). DNA Origami Route for Nanophotonics. ACS Photonics.

[166] Giljohann D.A., Seferos D.S., Prigodich A.E., Patel P.C., Mirkin C.A. (2009). Gene Regulation with Polyvalent SiRNA−Nanoparticle Conjugates. J. Am. Chem. Soc..

[167] Chou L.Y.T., Zagorovsky K., Chan W.C.W. (2014). DNA Assembly of Nanoparticle Superstructures for Controlled Biological Delivery and Elimination. Nat. Nanotechnol..

[168] Rosi N.L., Giljohann D.A., Thaxton C.S., Lytton-Jean A.K.R., Han M.S., Mirkin C.A. (2006). Oligonucleotide-Modified Gold Nanoparticles for Intracellular Gene Regulation. Science.

[169] Jensen S.A., Day E.S., Ko C.H., Hurley L.A., Luciano J.P., Kouri F.M., Merkel T.J., Luthi A.J., Patel P.C., Cutler J.I. (2013). Spherical Nucleic Acid Nanoparticle Conjugates as an RNAi-Based Therapy for Glioblastoma. Sci. Transl. Med..

[170] Sita T.L., Kouri F.M., Hurley L.A., Merkel T.J., Chalastanis A., May J.L., Ghelfi S.T., Cole L.E., Cayton T.C., Barnaby S.N. (2017). Dual Bioluminescence and Near-Infrared Fluorescence Monitoring to Evaluate Spherical Nucleic Acid Nanoconjugate Activity in Vivo. Proc. Natl. Acad. Sci. USA.

[171] Lin Y.-W., Liu C.-W., Chang H.-T. (2009). DNA Functionalized Gold Nanoparticles for Bioanalysis. Anal. Methods.

[172] Liu Y., Liu X., Liu H., Wang J., Zhang Y., Zhao W., Zhou J. (2022). DNA-Gated N-CDs@SiO_2_ Nanoparticles-Based Biosensor for MUC1 Detection. ChemistrySelect.

[173] Blumenfeld C.M., Schulz M.D., Aboian M.S., Wilson M.W., Moore T., Hetts S.W., Grubbs R.H. (2018). Drug Capture Materials Based on Genomic DNA-Functionalized Magnetic Nanoparticles. Nat. Commun..

[174] Ye X., Shi H., He X., Yu Y., He D., Tang J., Lei Y., Wang K. (2016). Cu–Au Alloy Nanostructures Coated with Aptamers: A Simple, Stable and Highly Effective Platform for in Vivo Cancer Theranostics. Nanoscale.

[175] Di Z., Liu B., Zhao J., Gu Z., Zhao Y., Li L. (2020). An Orthogonally Regulatable DNA Nanodevice for Spatiotemporally Controlled Biorecognition and Tumor Treatment. Sci. Adv..

[176] Lozano R., Naghavi M., Foreman K., Lim S., Shibuya K., Aboyans V., Abraham J., Adair T., Aggarwal R., Ahn S.Y. (2012). Global and Regional Mortality from 235 Causes of Death for 20 Age Groups in 1990 and 2010: A Systematic Analysis for the Global Burden of Disease Study 2010. Lancet.

[177] Shi J., Kantoff P.W., Wooster R., Farokhzad O.C. (2017). Cancer Nanomedicine: Progress, Challenges and Opportunities. Nat. Rev. Cancer.

[178] Xu J., Tanabe S. (2019). Persistent Luminescence Instead of Phosphorescence: History, Mechanism, and Perspective. J. Lumin..

[179] Lin Q., Li Z., Yuan Q. (2019). Recent Advances in Autofluorescence-Free Biosensing and Bioimaging Based on Persistent Luminescence Nanoparticles. Chin. Chem. Lett..

[180] Liu F., Liang Y., Chen Y., Pan Z. (2016). Divalent Nickel-Activated Gallate-Based Persistent Phosphors in the Short-Wave Infrared. Adv. Opt. Mater..

[181] Wu S., Li Y., Ding W., Xu L., Ma Y., Zhang L. (2020). Recent Advances of Persistent Luminescence Nanoparticles in Bioapplications. Nano-Micro Lett..

[182] Zheng B., Chen H., Zhao P., Pan H., Wu X., Gong X., Wang H., Chang J. (2016). Persistent Luminescent Nanocarrier as an Accurate Tracker in Vivo for Near Infrared-Remote Selectively Triggered Photothermal Therapy. ACS Appl. Mater. Interfaces.

[183] Wu S., Li Y., Zhang R., Fan K., Ding W., Xu L., Zhang L. (2021). Persistent Luminescence-Polypyrrole Nanocomposite for Dual-Modal Imaging and Photothermal Therapy of Mammary Cancer. Talanta.

[184] Montaseri H., Kruger C.A., Abrahamse H. (2020). Recent Advances in Porphyrin-Based Inorganic Nanoparticles for Cancer Treatment. Int. J. Mol. Sci..

[185] Silva L.B., Castro K.A.D.F., Botteon C.E.A., Oliveira C.L.P., da Silva R.S., Marcato P.D. (2021). Hybrid Nanoparticles as an Efficient Porphyrin Delivery System for Cancer Cells to Enhance Photodynamic Therapy. Front. Bioeng. Biotechnol..

[186] Qindeel M., Sargazi S., Hosseinikhah S.M., Rahdar A., Barani M., Thakur V.K., Pandey S., Mirsafaei R. (2021). Porphyrin-Based Nanostructures for Cancer Theranostics: Chemistry, Fundamentals and Recent Advances. ChemistrySelect.

[187] Xue X., Lindstrom A., Li Y. (2019). Porphyrin-Based Nanomedicines for Cancer Treatment. Bioconjug. Chem..

[188] Montané X., Matulewicz K., Balik K., Modrakowska P., Łuczak M., Pérez Pacheco Y., Reig-Vano B., Montornés J.M., Bajek A., Tylkowski B. (2021). Present Trends in the Encapsulation of Anticancer Drugs. Phys. Sci. Rev..

[189] Kirar S., Chaudhari D., Thakur N.S., Jain S., Bhaumik J., Laha J.K., Banerjee U.C. (2021). Light-Assisted Anticancer Photodynamic Therapy Using Porphyrin-Doped Nanoencapsulates. J. Photochem. Photobiol. B Biol..

[190] Fakayode O.J., Kruger C.A., Songca S.P., Abrahamse H., Oluwafemi O.S. (2018). Photodynamic Therapy Evaluation of Methoxypolyethyleneglycol-Thiol-SPIONs-Gold-Meso-Tetrakis(4-Hydroxyphenyl)Porphyrin Conjugate against Breast Cancer Cells. Mater. Sci. Eng. C.

[191] Bera K., Maiti S., Maity M., Mandal C., Maiti N.C. (2018). Porphyrin–Gold Nanomaterial for Efficient Drug Delivery to Cancerous Cells. ACS Omega.

[192] Melancon M.P., Stafford R.J., Li C. (2012). Challenges to Effective Cancer Nanotheranostics. J. Control. Release.

[193] Singh D., Dilnawaz F., Sahoo S.K. (2020). Challenges of Moving Theranostic Nanomedicine into the Clinic. Nanomedicine.

[194] Terentyuk G., Maslyyakova G., Suleymanova L., Kogan B., Khlebtsov B., Akchurin G., Makisimova I., Shantrokha A., Tuchin V. (2009). Tracking Gold Nanoparticles in the Body. J. Biomed. Opt..

[195] Ferdous Z., Nemmar A. (2020). Health Impact of Silver Nanoparticles: A Review of the Biodistribution and Toxicity Following Various Routes of Exposure. Int. J. Mol. Sci..

[196] Nowak-Jary J., Machnicka B. (2022). Pharmacokinetics of Magnetic Iron Oxide Nanoparticles for Medical Applications. J. Nanobiotechnol..

[197] Kundu P., Singh D., Singh A., Sahoo S.K. (2020). Cancer Nanotheranostics: A Nanomedicinal Approach for Cancer Therapy and Diagnosis. Anti-Cancer Agents Med. Chem..

[198] Ferreira M., Sousa J., Pais A., Vitorino C. (2020). The Role of Magnetic Nanoparticles in Cancer Nanotheranostics. Materials.

[199] Silva C.O., Pinho J.O., Lopes J.M., Almeida A.J., Gaspar M.M., Reis C. (2019). Current Trends in Cancer Nanotheranostics: Metallic, Polymeric, and Lipid-Based Systems. Pharmaceutics.

[200] Khandker S.S., Shakil S., Hossen S. (2020). Gold Nanoparticles; Potential Nanotheranostic Agent in Breast Cancer: A Comprehensive Review with Systematic Search Strategy. Curr. Drug Metab..

[201] Hadadian Y., Uliana J.H., Carneiro A.A.O., Pavan T.Z. (2021). A Novel Theranostic Platform: Integration of Magnetomotive and Thermal Ultrasound Imaging With Magnetic Hyperthermia. IEEE Trans. Biomed. Eng..

[202] Wang Y., Meng H.-M., Song G., Li Z., Zhang X.-B. (2020). Conjugated-Polymer-Based Nanomaterials for Photothermal Therapy. ACS Appl. Polym. Mater..

[203] Zhong X., Dai X., Wang Y., Wang H., Qian H., Wang X. (2022). Copper-based Nanomaterials for Cancer Theranostics. WIREs Nanomed. Nanobiotechnol..

